# Aldolase A accelerates hepatocarcinogenesis by refactoring c-Jun transcription

**DOI:** 10.1016/j.jpha.2024.101169

**Published:** 2024-12-16

**Authors:** Xin Yang, Guang-Yuan Ma, Xiao-Qiang Li, Na Tang, Yang Sun, Xiao-Wei Hao, Ke-Han Wu, Yu-Bo Wang, Wen Tian, Xin Fan, Zezhi Li, Caixia Feng, Xu Chao, Yu-Fan Wang, Yao Liu, Di Li, Wei Cao

**Affiliations:** a Department of Pharmacy, School of Chemistry & Pharmacy, Northwest A&F University, Yangling, Shaanxi, 712100, China; b Shaanxi Key Laboratory of Natural Products & Chemical Biology, Northwest A&F University, Yangling, Shaanxi, 712100, China; c Key Laboratory of Gastrointestinal Pharmacology of Chinese Materia Medica of the State Administration of Traditional Chinese Medicine, School of Pharmacy, Air Force Medical University, Xi’an, 710032, China; d Department of Chinese Materia Medica and Natural Medicines, School of Pharmacy, Air Force Medical University, Xi’an, 710032, China; e The Second Affiliated Hospital of Shaanxi University of Traditional Chinese Medicine, Xianyang, Xi'an, 712046, China; f Life Science Research Core Service Platform, Northwest A&F University, Yangling, Shaanxi, 712100, China; g National Human Genetic Resources Sharing Service Platform (2005DKA21300), Shanghai, 201023, China; h Shanghai Engineering Technology Center for Molecular Medicine, Shanghai, 201023, China

**Keywords:** Hepatocellular carcinoma, Glycolysis, c-Jun, Nuclear localization, Transcriptional regulation

## Abstract

Hepatocellular carcinoma (HCC) expresses abundant glycolytic enzymes and displays comprehensive glucose metabolism reprogramming. Aldolase A (ALDOA) plays a prominent role in glycolysis; however, little is known about its role in HCC development. In the present study, we aim to explore how ALDOA is involved in HCC proliferation. HCC proliferation was markedly suppressed both *in vitro* and *in vivo* following *ALDOA* knockout, which is consistent with *ALDOA* overexpression encouraging HCC proliferation. Mechanistically, *ALDOA* knockout partially limits the glycolytic flux in HCC cells. Meanwhile, ALDOA translocated to nuclei and directly interacted with c-Jun to facilitate its Thr93 phosphorylation by P21-activated protein kinase; *ALDOA* knockout markedly diminished c-Jun Thr93 phosphorylation and then dampened c-Jun transcription function. A crucial site Y364 mutation in ALDOA disrupted its interaction with c-Jun, and Y364S ALDOA expression failed to rescue cell proliferation in *ALDOA* deletion cells. In HCC patients, the expression level of ALDOA was correlated with the phosphorylation level of c-Jun (Thr93) and poor prognosis. Remarkably, hepatic ALDOA was significantly upregulated in the promotion and progression stages of diethylnitrosamine-induced HCC models, and the knockdown of *Aldoa* strikingly decreased HCC development *in vivo*. Our study demonstrated that ALDOA is a vital driver for HCC development by activating c-Jun-mediated oncogene transcription, opening additional avenues for anti-cancer therapies.

## Highlights

• ALDOA knockout significantly inhibits HCC cell proliferation without affecting normal liver cells.
• ALDOA knockout partially limits the glycolytic flux in HCC cells.
• ALDOA interacts with c-Jun and regulates its transcriptional activity by affecting c-Jun (Thr93) phosphorylation in HCC cells.
• ALDOA binds to c-Jun to enhance the transcription of the *ALDOA* gene.
• AAV8-mediated liver *Aldoa* knockdown in the HCC mouse model decreases HCC development.

## Graphical abstract

**Figure:**
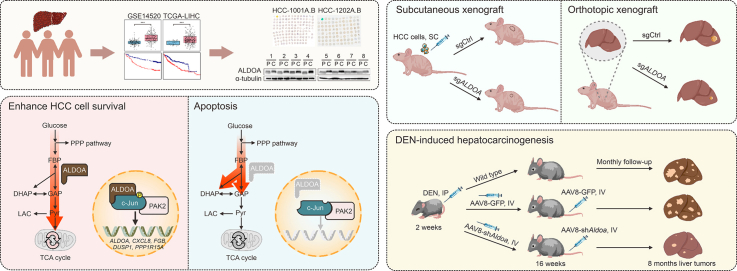

## 1 Introduction

Metabolic reprogramming is a hallmark of malignancy [1]. It is commonly acknowledged that a distinctive character of metabolic reprogramming in cancer cells is the Warburg effect, which is defined as the preference for aerobic glycolysis beyond oxidative phosphorylation at physiological oxygen concentrations [2]. This metabolic reprogramming facilitates the provision of necessary metabolites for rapid growth, making it a crucial factor in the development and progression of cancer [3]. Among various types of cancer cells, hepatocellular carcinoma (HCC), as a primary liver cancer, exhibits the most extensive reprogramming of glucose metabolism and prominently expresses a wide array of glycolytic enzymes [4]. Similar to all cancers, HCC arises from uncontrolled growth of mature hepatocytes due to the gradual accumulation of genetic and epigenetic modifications in liver cells, accompanied by significant metabolic alterations [5]. Among these changes, elevated glycolysis plays a crucial role in promoting malignancy by facilitating the production of macromolecules (such as nucleotides, amino acids, and fatty acids) and adenosine triphosphate (ATP) for biomass expansion, while also impacting microenvironmental pH [6], disrupting reactive oxygen species equilibrium, and modulating chromatin remodeling. Recent research has revealed that the metabolic shift in liver samples from patients with HCC is a result of the activation of oncogenes or the loss of tumor suppressors, which subsequently affect the expression of glucose metabolic enzymes in the glycolytic pathway [7]. For example, a significant alteration observed in HCC is the upregulation of hexokinase 2, the initial enzyme in glycolysis, promoting the conversion of glucose [8]. Interestingly, in addition to canonical enzyme functions, there is growing evidence that many metabolic enzymes can stimulate tumor cell proliferation through noncanonical functions (moonlighting functions) [9]. Whether based on canonical or noncanonical function, the current theoretical framework posits that the regulation of HCC cells could be achieved by targeting glycolytic enzymes, which show selective expression in normal hepatocytes [7]. Consequently, laboratories across the globe have been actively investigating inhibitors of metabolic enzymes that could potentially serve as anticancer drugs [6]. However, the precise biological significance of the heightened expression of glycolytic enzymes in HCC compared to hepatocytes is still not well understood.

Aldolase plays a crucial role in the glycolytic pathway by catalyzing the reversible cleavage of fructose 1,6-bisphosphate (FBP) to produce glyceraldehyde 3-phosphate (GAP) and dihydroxyacetone phosphate (DHAP). Under physiological conditions, aldolase A (ALDOA) acts as a glucose sensor through AMP-activated protein kinase (AMPK), mediated by the v-ATPase complex [10] and mobilization from F-actin skeletons. This regulatory mechanism of glycolysis leads to enhanced metabolic efficiency. Multiple studies have consistently demonstrated the overexpression of ALDOA in cancer cells. This protein has been found to exacerbate the invasion and migration abilities of cancer cells, and more significantly, it plays a crucial role in promoting proliferation in various types of cancer, such as lung cancer [11], highly metastatic pancreatic cancer [12], and cervical adenocarcinoma [13]. ALDOA is subject to regulation by hypoxia-inducible factor 1-alpha (HIF-1α) and further regulates metabolic events crucial for most tumors' development [14]. The upregulation of ALDOA expression may be associated with the hypoxia response in HCC. Furthermore, loss-of-function genetic screening has identified ALDOA as a critical driver for HCC growth in hypoxic conditions [15]. However, in comparison to HCC, other primary tumors exhibit a more pronounced hypoxic state [16], as HCC is a hypervascular tumor that benefits from ample oxygen supply via the portal vein [17]. To date, the specific functions of ALDOA overexpression in HCC and whether inhibiting ALDOA could effective treatment of HCC are yet to be confirmed.

In this study, ALDOA was selected as a proof-of-principle target to investigate the role of upregulated glycolytic enzymes in HCC. The tumor-promoting role of ALDOA in HCC was confirmed through both *in vitro* and *in vivo* experiments. Metabolic flow analysis (MFA) and the determination of glycolysis products provided further evidence that the knockout of *ALDOA* disrupted intracellular glucose metabolism. Notably, our findings demonstrated that in HCC cells, the overexpression of ALDOA could translocate from the cytoplasm to the nucleus, where it bound to c-Jun and facilitated the phosphorylation of c-Jun at the Thr93 site by P21-activated kinase 2 (PAK2). c-Jun^T93^ phosphorylation enhanced the transcription function of c-Jun and triggered a series of cellular proliferation events. Furthermore, ALDOA gene therapy mediated by the adeno-associated virus (AAV) significantly suppresses the incidence and progression of HCC in mice. Overall, our findings suggest that ALDOA plays a crucial role in HCC development, and targeting ALDOA is a promising strategy for HCC treatment.

## 2 Materials and methods

### 2.1 Cell culture

THLE2, HHCC, HepG2, Huh-7, MHCC97L, MHCC97H, and HCCLM3 cells were obtained from the Cell Bank of Type Culture Collection of the Chinese Academy of Sciences, Shanghai Institute of Cell Biology (Shanghai, China). HEK293T (ATCC accession no. CRL-3216) was obtained from Vigene Biosciences (Jinan, China). All cells were authenticated by DNA fingerprinting with small tandem repeat profiling and validated to be free of mycoplasma contamination by using MycoFluor™ mycoplasma detection kits (Thermo Fisher Scientific Inc., Waltham, MA, USA).

HHCC cells were cultured in Roswell Park Memorial Institute 1640 medium (RPMI1640; Gibco, Grand Island, NY, USA) supplemented with 10% (*v/v*) fetal bovine serum (FBS; Merck, St Louis, MO, USA) and 1% (*v/v*) penicillin-streptomycin (Pen/Strep; Solarbio, Beijing, China). THLE2, HepG2, Huh-7, MHCC97L, MHCC97H, HCCLM3, and HEK293T cells were cultured in high glucose Dulbecco’s Modified Eagle Medium (DMEM; Gibco) medium supplemented with 10% (*v/v*) FBS and 1% (*v/v*) Pen/Strep. The cells were cultured at 37 °C in a humidified incubator with 5% CO_2_.

### 2.2 Mice

Male C57BL/6 mice were obtained from the Laboratory Animal Center of the Fourth Military Medical University (FMMU). Male athymic BALB/c nude mice were purchased from Vital River Laboratories (Beijing, China). All mice were born and maintained under pathogen-free conditions. Animal care and handling were performed in accordance with the recommendations in the Guide for the Care and Use of Laboratory Animals of the National Institutes of Health. All animal studies were approved by the Animal Care Committee of Northwest A&F University (Approval number: 2452017335-1).

### 2.3 Clinical specimens

HCC and the corresponding para-cancerous tissues from eight patients diagnosed with HCC between June 2009 and May 2016 were obtained from the National Human Genetic Resources Sharing Service Platform (2005DKA21300; Shanghai, China) for Western blot analysis. The patient case codes are D19A1206, D19A1251, D19A1262, D19A2737, D19A2739, D19A2744, D19A3930, and D19A3936. Human HCC tumor tissue microarrays (no. HCC-1001A.B and HCC-1202A.B) were obtained from the Xijing Hospital of FMMU for morphological and immunohistochemical analyses. Human HCC tissue samples were collected after obtaining written informed consent from the patients in accordance with the ethical guidelines of the National Human Genetic Resources Sharing Service Platform or the Institutional Review Board of Xijing Hospital of FMMU.

### 2.4 Lentivirus production and transduction

For knockout of *ALDOA* in cells, all single guide RNAs were designed using the clustered regularly interspaced short palindromic repeats (CRISPR) design tool (http://crispr.mit.edu/) and cloned into the LentiCRISRRv2-puro plasmid, constructed by Vigene Biosciences (Jinan, China). The *ALDOA* CRISPR and CRISPR-associated protein 9 plasmid or negative control empty vector was transfected into THLE2, HepG2, and HCCLM3 cells to knockout the expression of *ALDOA*. Correspondingly, human *ALDOA* (GenBank® accession no. CR536528) full-length complementary DNA (cDNA) was synthesized and cloned into the pLV-EGFP (2A) lentiviral puro vector and then transfected into HepG2 cells to overexpress *ALDOA*, designated as HepG2-*ALDOA*. The control vector-transfected cells were designated as HepG2-vector. After transduction, the positive cells were selected with puromycin (5 mg/mL; Thermo Fisher Scientific Inc.) for up to two weeks. The protein expression of ALDOA was tested by Western blot analysis to confirm successful knockout or overexpression.

### 2.5 Cell proliferation assay

The cell proliferation was measured by a Cell Counting Kit-8 (Beyotime, Shanghai, China). Cells were trypsinized to prepare cell suspensions and seeded in 96-well plates at a density of 2,000 per well. At different time points (0–96 h) after plating, Cell Counting Kit-8 solution was added to each well and measured at 450 nm using a microplate reader (Tecan spark, Tecan, Männedorf, Switzerland).

### 2.6 Clone formation assay

Cells were seeded in 6-well plates at a density of 300 per well. The cells were cultured until day 14 and then stained with crystal violets (Beyotime). Cell clones were then counted under a light microscope. A cell clone was defined to include at least 50 cells.

### 2.7 Flow cytometry assay

The cell cycle distribution was measured by a cell cycle analysis kit (4A Biotech Co., Ltd., Beijing, China). Cells were trypsinized into cell suspension, fixed with 95% ethanol overnight at 4 °C, and washed twice with phosphate-buffered saline (PBS; Solarbio). The samples were then treated with 0.025 μg/μL ribonuclease for 30 min at 37 °C, stained with propidium iodide for 30 min at 37 °C in a dark area, and then determined by a flow cytometer (Becton Dickinson, Franklin City, CA, USA).

### 2.8 Cell invasion and migration assay

Cell migration was determined by wound healing assay. Cells were seeded in 24-well plates, then incubated until reaching 90% confluence. Cells were serum-starved for 24 h, and then a pipette tip was used to create a linear wound. The wound was immediately photographed at 0 h and observed at 24 and 48 h. The invasion of cells was assessed by Transwell assay. Matrigel (#354234, Corning, Corning, NY, USA) was used to coat the surface of the upper chamber, and then cells were seeded into the upper chamber at a density of 1 × 10^5^ per well and incubated for 48 h. After the cells were fixed with cold methanol, they were stained with 0.1% crystal violet and counted.

### 2.9 Aldolase enzymatic assay

The aldolase enzymatic activity assay was performed based on monitoring nicotinamide adenine dinucleotide plus hydrogen levels via a coupled enzymatic reaction using triosephosphate isomerase and α-glycerol phosphate dehydrogenase. Cells were seeded in 6-well plates and incubated until reaching 80% confluence. The enzymatic activity assay was determined using an aldolase enzyme activity detection kit (Genmed Scientifics, Shanghai, China) following the manufacturer’s instructions. Briefly, after the reaction mixtures and cell lysate were added to a 96-well quartz plate, a decrease in absorbance at 340 nm was monitored in 5 min intervals with a microplate reader (Tecan spark, Tecan).

### 2.10 MFA

Cells (2 × 10^7^/sample) were seeded in 6-cm plates and incubated until reaching 80% confluence in complete medium. The medium was replaced with glucose-free DMEM medium supplemented with 5 mM [U–^13^C6] glucose (Cambridge Isotope Laboratories, Andover, MA, USA) or glutamine-free DMEM medium supplemented with 4 mM [U–^13^C5] glutamine (Cambridge Isotope Laboratories). Stable isotope-tracing analysis was performed at 6 or 20 h post-treatment, as previously described [8,18]. Briefly, the medium was removed, and cells were quenched with a quencher (60% methanol and 0.85% ammonium bicarbonate, pH 7.4) for 15 min at −80 °C. The cell samples were processed by 5 cycles of 2 min ultra-sonication and 2 min intervals in an ice-water bath. After centrifugation at 16,000 *g* for 15 min, the supernatant was transferred to a new tube. The metabolite samples analyzed by ultra-high-performance liquid chromatography high-resolution tandem mass spectrometry (UHPLC-HRMS/MS) were desiccated under nitrogen gas flow and re-dissolved in a 50% acetonitrile aqueous solution. Metabolites were analyzed with a BEH Amide column (2.1 mm × 100 mm, 1.7 μm; Waters, Milford, MA, USA) using an Ultimate 3000 UHPLC system (Thermo Fisher Scientific Inc.) coupled to a Q Exactive™ Hybrid Quadrupole-Orbitrap™ mass spectrometer (Thermo Fisher Scientific Inc.) in negative ion mode with the heated electrospray ionization ion source. Correspondingly, metabolite samples analyzed using gas chromatography-mass spectrometry were incubated with methoxyamine hydrochloride in pyridine at 37 °C for 90 min and then derivatized in N-methyl-N-(tert-butyldimethylsilyl)- trifluoroacetamide and 1% tert-butyldimetheyl-chlorosilane at 55 °C for 60 min. The metabolites were analyzed using an Agilent 7890A gas chromatography system (Agilent Technologies, Santa Clara, CA, USA) coupled with an Agilent 5975C inert MSD system, using an OPTIMA® 5 MS Accent fused-silica capillary column (30 m × 0.25 mm × 0.25 μm; MACHEREY-NAGEL, Düren, Germany). Mass isotopomer distributions were obtained by integration and corrected for natural isotope abundances.

### 2.11 The determination of metabolic products of glycolysis

The glycolytic products were measured by a liquid chromatography-mass spectrometry (LC-MS) approach, as previously described [19]. Briefly, cells were collected and added with 1 mL methanol/water (1:1, *v/v*; −50 °C) to stop cellular metabolism. The samples were centrifuged at 4 °C and the supernatant was removed. The solid cell pellet was resuspended by vortexing in a 500 μL mixture of methanol and water (1:1, v*/v*; −20 °C). After the addition of 0.3 M KOH (dissolved in 25% ethanol), the mixture was neutralized by adding 40 μL of glacial acetic acid. Cell debris was removed by centrifugation at −20 °C. All metabolite samples were performed on a 3000 Exion LC System (SCIEX, Singapore City, Singapore) which was coupled with a 5500 QTRAP instrument (AB SCIEX, Singapore City, Singapore). The samples were injected into a 100 mm × 2.1 mm HILIC column (Shimadzu, Tokyo, Japan). The mobile phase consisted of solvent A (5 mM ammonium acetate in water) and solvent B (5 mM ammonium acetate in acetonitrile) with the following linear gradient elution steps: 2 min (5% A–95% B)–7 min (40% A–60% B)–10 min (95% A–5% B). The flow rate was kept at 300 μL/min. The MS operated in the full scan-selected reaction monitoring mode using an ion spray voltage of −4.5 kV, entrance potential of −10 V, and auxiliary gas at 550 °C. All data were acquired and evaluated via Analyst software (AB SCIEX, Singapore City, Singapore).

### 2.12 Seahorse assay

Glycolysis test was performed with an XF24 extracellular flux analyzer (Agilent Technologies) according to the manufacturer’s instructions. 5 × 10^4^ cells were seeded in 24-well plates overnight. Extracellular acidification rate (ECAR) was measured using Seahorse XF Glycolysis Stress Test Kits (Agilent Technologies), as previously described. Briefly, the ECAR was measured by an extracellular flux analyzer with sequential injection of 10 mM glucose, 1 μM oligomycin, and 50 mM 2-deoxy-d-glucose. After measurement, cells were trypsinized and counted with a Coulter Counter (Beckman Coulter, Fullerton, CA, USA). The values were normalized by the total number of cells.

### 2.13 RNA sequencing (RNA-seq) and analysis

The gene expression differences in *ALDOA* knockout HCCLM3 cells and control cells were determined by RNA-seq. DNA library preparation and sequencing were conducted by KangChen Bio-tech Co., Ltd. (Shanghai, China). Briefly, the sequencing libraries were generated from 2 μg of total RNA using stranded RNA-Seq Library Prep Kit (Illumina, San Diego, CA, USA). Sequencing was performed using the Illumina HiSeq4000 platform. Image analysis and base calling were performed using Solexa pipeline V1.8 (Off-Line Base Caller software, version 1.8). The gene & transcript expression levels (fragments per kilobase per million mapped reads (FPKM) value) and any significant changes were calculated using Ballgown (version 2.8.4). Enrichment analysis of the differential expressed genes was performed using an online analysis tool, String (https://www.string-db.org/).

Gene set enrichment analysis was used to explore the biological process and signaling pathways associated with the expression of *ALDOA* in the HCCLM3 cells. Enrichment analysis was carried out using positional, curated, and ontology gene sets. The number of gene set permutations was 1,000 times for each analysis. The gene set was considered significantly enriched if the *P* value <0.05 and false discovery rate *q* value <0.05.

### 2.14 Co-immunoprecipitation (co-IP) assay

Co-IP assays were carried out using Pierce™ Co-Immunoprecipitation kits (Thermo Fisher Scientific Inc.) following the manufacturer’s instructions. Briefly, 2–6 μg anti-ALDOA (#11217-1-AP, Proteintech, Wuhan, China), anti-c-Jun (#15683, Cell Signaling Technologies, Beverly, MA, USA), anti-MYC Tag (#60003-2-Ig, Proteintech), anti-FLAG Tag (#20543-1-AP, Proteintech), rabbit IgG (#A7016, Beyotime), or mouse IgG (#A7028, Beyotime) antibodies were incubated with 20 μL protein A/G sepharose at room temperature for 1 h. Cells were lysed in ice-cold lysis buffer supplemented with 1 mM phenyl methane sulfonyl fluoride. After centrifuging, the lysates were quantified for protein concentrations and incubated with control IgG- or antibody-bound sepharose at 4 °C overnight. The samples were washed with lysis buffer, denatured, and detected by western blotting.

### 2.15 Immunofluorescence assay

Cells were cultured on micro cover glasses precoated with poly-l-lysine in 24-well plates and fixed with 4% paraformaldehyde. The optimal cutting temperature compound (Sakura, Torrance, CA, USA) embedded tissues were cryosectioned into 10-μm-thick sections. Antigen retrieval was performed with citrate buffer (pH 6.0) by heating in a microwave. After permeabilizing with 0.2% Triton X-100 for 15 min and blocking with 10% goat serum, the slides were incubated with primary antibodies including anti-ALDOA (C-10) (1: 300, #sc-390733, Santa Cruz Biotechnology, Dallas, TX, USA) and anti-c-Jun (60A8) (1:400, #15683, Cell Signaling Technologies) for 2 h at room temperature. The slides were washed with PBS and then incubated with fluorescein isothiocyanate or sulfo-cyanine 3 label-conjugated secondary antibodies for 1 h at room temperature. After several washes with PBS, the slides were treated with an anti-fade mounting medium containing 4′,6-diamidino-2-phenylindole (DAPI) or not. The images were acquired using confocal microscopy (FV3000, Olympus, Tokyo, Japan).

### 2.16 Proximity ligation assay (PLA)

PLA were carried out using Duolink PLA kit (Sigma-Aldrich, St. Louis, MO, USA) following the manufacturer’s instructions. Briefly, THLE2 and HCCLM3 cells were cultured on micro cover glasses in 24-well plates and fixed with 4% paraformaldehyde. Following fixation, cells were permeabilized using 0.1% Triton X-100 for 15 min and then blocked with Duolink blocking buffer for 30 min. The cells were then incubated overnight at 4 °C with primary antibodies diluted in Duolink antibody diluent. After washing, the cells were incubated with the appropriate Duolink secondary antibodies for 1 h at 37 °C. Subsequently, the ligation and amplification steps of the PLA were conducted using Duolink *in situ* Detection Reagents FarRed (Sigma-Aldrich). The cells were treated with an anti-fade mounting medium containing DAPI and imaged utilizing a confocal microscope (FV3000, Olympus). The quantification of PLA spots and nuclei was performed on a single image using the “analyze particle” function of an IJM language macro in Fiji/Image J software (National Institutes of Health, Bethesda, MD, USA).

### 2.17 Docking studies

The crystal structure of ALDOA (PDB ID. 1ALD) was obtained from the Protein Data Bank database. The 3D model of the N-terminal domain of human c-Jun (GenBank® accession no. BC068522) was built using the Discovery Studio 4.0 software (Accelrys, Inc., San Diego, CA, USA). The interaction between ALDOA and the N-terminal domain of human c-Jun was studied by protein-protein docking tools using Z dock protocol in Discovery Studio as previously described [20].

### 2.18 Construction and transfection of human ALDOA and c-Jun mutant vectors

Full-length cDNAs of human *ALDOA* (GenBank® accession no. CR536528) and *JUN* (GenBank® accession no. BC068522) were amplified using standard polymerase chain reaction (PCR) techniques and inserted into the pcDNA3.1 (+) expression vectors with the indicated C-terminal fusion FLAG or MYC tags. Mutants of E35D, K42N, D129V, K230A, and Y364S ALDOA, as well as Y10S c-Jun, were constructed with the Site-Directed Mutagenesis kit (Thermo Fisher Scientific Inc.) following the manufacturer’s instructions. All recombinants were verified by sequencing. HEK293T cells were seeded in 6-well plates and incubated until reaching 60% confluence. The recombinant plasmids were transfected into HEK293T cells using the LipoFiter™ liposomal transfection reagent (Hanbio Biotechnology, Shanghai, China) following the manufacturer’s instructions. At 48 or 72 h after transfection, cells were harvested for further assays.

### 2.19 The xenograft mouse model

For subcutaneous xenograft models, 6-week-old BALB/c nude mice were subcutaneously injected in the right flanks with 4 × 10^6^ different HCC cells in 200 μL medium. Tumor sizes were measured with a vernier caliper every 4 days and tracked manually for 4 weeks. Tumor volumes (V) were calculated using the following equation: *V* = 0.5 × length × width^2^. All mice were sacrificed 28 days later through inhaled ether. After excising and weighing, tumors were photographed and kept for further analysis.

For the orthotopic xenograft model, 5 × 10^6^ cells were subcutaneously injected in the right flank region of nude mice. Subcutaneous tumor tissues were removed upon reaching a size of approximately 1 cm in diameter. Subsequently, they were dissected into 1 mm^3^ sections. The dissected tumor tissues were then orthotopically implanted into the right hepatic lobe of eight 6-week-old BALB/c nude mice. The mice were sacrificed after 42 days of implantation, and tumor volumes were calculated. After excising and weighing, the livers and lungs were photographed, fixed in 4% paraformaldehyde, embedded in paraffin, and then serially sectioned for histopathological examination.

### 2.20 Diethylnitrosamine (DEN)-induced hepatocarcinogenesis and virus administration

Hepatocarcinogenesis was induced by a single high-dose (40 mg/kg body weight) intraperitoneal injection of DEN (Aladdin, Shanghai, China) in male C57BL/6 mice at the age of 14 days. Development of liver tumors was followed up at 1, 2, 3, 4, 5, 6, 7, and 8 months after DEN exposure, and the mice were sacrificed by inhaling ether. After excising and weighing, the livers were photographed and fixed in 4% paraformaldehyde to be used for hematoxylin and eosin (H&E) staining and immunohistochemical analysis or lysed for Western blot analysis.

To examine whether *Aloda* knockdown in the liver could suppress DEN-induced hepatocarcinogenesis, we constructed a liver-specific adeno-associated virus based on serotype 8 (AAV8) containing the *Aldoa* short-hairpin RNA (shRNA) coding sequence, as previously described [21]. The duplex oligonucleotides shRNA construct directed against *Aldoa* was cloned into pAV-U6-GFP using the BamHI and HindIII restriction sites (Hanbio Biotechnology, Shanghai, China). Male C57BL/6 mice at the age of 14 days were intraperitoneally injected with DEN (40 mg/kg body weight) or normal saline (control group). Twenty DEN-exposed and control mice received a single intravenous injection of 1 × 10^12^ vector genomes of AAV8/pAV-U6-GFP-sh*Aldoa* or AAV8/pAV-U6-GFP at 2 and 16 weeks after DEN treatment, respectively. The knockdown sequences were confirmed both *in vitro* and *in vivo*. At 8 months after DEN exposure, the mice were sacrificed by inhaling ether. The body and liver weights of the animals were measured, and total serum, livers, lungs, muscles, and hearts were collected. Grossly visible liver tumors were measured with calipers and counted. The formula of max tumor volume is 0.5 × length × width^2^. Tissues were either fixed in 4% paraformaldehyde or optimal cutting temperature compound (Sakura) for histological analysis or stored at −80 °C following the standard protocols.

### 2.21 Immunohistochemistry (IHC) analysis

Tissue sections embedded in paraffin were sectioned, deparaffinized, and rehydrated. After the endogenous peroxidase was inactivated with 3% hydrogen peroxide, the samples were subjected to antigen retrieval performed in 10 mM citrate buffer (pH 6.0). The slides were washed in PBS, incubated in blocking buffer (5% bovine serum albumin (BSA)) for 1 h and incubated with the anti-ALDOA (1:200, #sc-377058, Santa Cruz Biotechnology) or anti-Ki67 (1:600, Servicebio, Wuhan, China) antibody, at room temperature for 2 h. Subsequently, the slides were washed in PBS and incubated with the biotin-labeled antibodies (SA1021 and SA1022, Boster Biological Technology Co. Ltd, Wuhan, China) at room temperature for 1 h. The sections were incubated with the streptavidin-biotin complex at 37 °C for 30 min, stained with 3,3′-diaminobenzidine, and then counterstained with hematoxylin. The slices were photographed with an Olympus IX73 microscope (Olympus) or scanned with a full-field digital slice scanner Pannoramic DESK (3D Histech, Budapest, Hungary).

The average gray value of ALDOA positive cells and the percentage of positive area in the field analyzed were measured using the plugin IHC Profiler in Fiji/Image J software on whole tissue sections. The intensity of immunostaining was divided into four grades (intensity scores): high positive (score 3), positive (score 2), low positive (score 1), and negative (score 0). Scores 3 and 2 represent high ALDOA expression, whereas score 1 and score 0 represent low ALDOA expression. The average gray value of Ki67 positive cells and the percentage of positive area in the field analyzed were measured using Image-Pro Plus software (Media Cybernetics, Rockville, MD, USA). The individual who analyzed the histologic samples was blinded to the treatment. Each data point is represented by 12 images from 4 animals.

### 2.22 Western blotting

Total protein lysates were obtained by lysing cells or tissues in radioimmunoprecipitation assay buffer containing phenylmethanesulfonyl fluoride and protease inhibitor cocktail. Extracted proteins were quantified using the BCA protein assay kit (Beyotime). Western blotting was performed according to the standard protocol. Briefly, equal amounts of protein were separated by sodium dodecyl sulfate-polyacrylamide gel electrophoresis and transferred to nitrocellulose membranes followed by blocking with 5% non-fat milk in PBS-0.05% Tween-20 (PBS-T). The membranes were incubated with the primary antibodies, washed with PBS-T, and then incubated with horseradish peroxidase-linked species-specific secondary antibodies. The signals were visualized by a Tanon-5200 Imaging System (Tanon Science and Technology Co. Ltd., Shanghai, China), and protein quantitation was performed by Fiji/Image J software. Primary antibodies used for Western blot analysis were as follows: anti-ALDOA (1:2000, #11217-1-AP, Proteintech), anti-c-Jun (1:1000, #66313-1-Ig, Proteintech), anti-c-Jun (phosphor Ser73) (1:1000; #3270, Cell Signaling Technologies), anti-AP-1 (phosphor Thr91) (1:300, #ABP54979, Abbkine, Wuhan, China), anti-AP-1 (phosphor Thr93) (1:300, #ABP50264, Abbkine), anti-AP-1 (phosphor Ser243) (1:300, #ABP54978, Abbkine), anti-FOS (1:1000, #66590-1-Ig, Proteintech), anti-α-tubulin (1:2000, #66031-1-Ig, Proteintech), anti-PAK2 (1:1000, #19979-1-AP, Proteintech), anti-MYC Tag (1:1000, #60003-2-Ig, Proteintech), anti-FLAG Tag (1:1000, #20543-1-AP, Proteintech), anti-Erk1/2 (1:1000, #4695, Cell Signaling Technologies), anti-p38 (1:1000, #8690, Cell Signaling Technologies), anti-SAPK/JNK (1:1000, #9252, Cell Signaling Technologies), anti-Erk1/2 (Thr202/Thr204) (1:1000, #4370, Cell Signaling Technologies), anti-p38 (Thr180/Tyr182) (1:1000, #4511, Cell Signaling Technologies), anti-SAPK/JNK (Thr183/Tyr185) (1:1000, #4668, Cell Signaling Technologies), and anti-DUSP1 (1:1000, #ab1351, Abcam, Cambridge, MA, USA).

### 2.23 Quantitative real-time PCR (qPCR)

Total RNA was extracted using the Trizol reagent (Tiangen Biotech Co. Ltd, Beijing, China) according to the manufacturer’s instructions and reversely transcribed into cDNA using Hifair® III 1st Strand cDNA Synthesis kits (Yeasen Biotechnology Co. Ltd., Shanghai, China) according to the manufacturer’s protocol. qPCR was performed using Hieff® qPCR SYBR Green Master Mix kits (Yeasen Biotechnology Co. Ltd.) on QuantStudio 3 Real-Time PCR System (Applied Biosystems, Foster City, CA, USA). The primers used for qPCR amplification were provided in Table S1. The program was as follows: an initial denaturation step at 95 °C for 30 s, followed by 40 cycles of denaturation at 95 °C for 10 s, annealing at 60 °C for 20 s, and extension at 72 °C for 20 s. A melting curve analysis of each sample was used to check the specificity of amplification. The messenger RNA (mRNA) expression levels were normalized to glyceraldehyde-3-phosphate dehydrogenase (*GAPDH*) and the relative expressions of target genes were calculated using the 2^−ΔΔC^^T^method.

### 2.24 Lactate production and glucose consumption

Cells were seeded in 24-well plates at a density of 5 × 10^5^ per well and incubated overnight. The lactate concentrations and glucose consumption in the culture supernatant were determined using a lactate assay kit (Solarbio) and a glucose consumption kit (Leagene Biotechnology, Beijing, China) according to the manufacturer’s protocols, respectively. Serum lactate levels were measured using a lactate assay kit (Jiancheng Bioengineering Institute, Nanjing, China).

### 2.25 Measurement of ATP

Cells were seeded in 6-well plates at a density of 2 × 10^6^ per well and incubated overnight. The cells were washed in PBS, collected, and then lysed to release ATP. The extracted proteins were quantified using the BCA protein assay kit (Beyotime). The ATP levels were measured using an ATP assay kit (Beyotime) according to the manufacturer’s protocol.

### 2.26 Bioinformatics analysis

The gene expression data, clinical significance, and follow-up information of the patients were obtained from the Cancer Genome Atlas Liver Hepatocellular Carcinoma (TCGA-LIHC) data portal (http://tcga-data.nci.nih.gov/tcga/), which covered 50 normal liver tissues and 369 HCC tissues. In addition, the GSE14520 dataset was directly accessed through the Gene Expression Omnibus (GEO) website (http://www.ncbi.nlm.nih.gov/geo), which included microarray expression data from 220 normal cases and 225 HCC cases.

To investigate the clinical implication of *ALDOA* expression, HCC patients were classified into two groups (high vs. low expression) based on the optimal cutoff values of the gene expression level, as determined by the “surv_cutpoint” function of the “survminer” R package (http://www.R-project.org/). Survival analysis, receiver operating characteristic (ROC) analysis, and univariate or multivariate Cox proportional hazards regression analysis were performed through R software environment according to the high- and low-*ALDOA* expressions in HCC patients.

To facilitate the prognosis evaluation of HCC patients, a nomogram was constructed based on prognostic clinical factors and *ALDOA* gene expression using the “rms” R package. Then, the predictive power of the nomogram was systematically assessed by the concordance index and calibration curves. The GSM935364 dataset was downloaded from the GEO website to find genes regulated by c-Jun, including data of chromatin immunoprecipitation sequencing (ChIP-Seq) in HepG2. The target genes were verified based on the chromatin immunoprecipitation (ChIP) peak annotation, comparison, and visualization by the “ChIPseeker” R package as previously described [22].

### 2.27 Serum assay

The serum levels of alanine aminotransferase (ALT), aspartate transaminase (AST), and glucosylated serum protein (GSP) as well as blood glucose level were measured with an automatic biochemical analyzer (Rayto, Shenzhen, China) using different commercial test kits (Nanjing Jiancheng Bioengineering Institute, Nanjing, China) according to the manufacturer’s instructions.

### 2.28 Histopathological observation

The tumor, liver, and lung tissues were fixed in 4% paraformaldehyde, embedded in paraffin, and then serially sectioned at 4 μm thickness for H&E staining. The images were acquired using an Olympus IX73 microscope (Olympus).

### 2.29 Statistical analysis

Data were presented as mean ± standard error of the mean (SEM), and all statistical tests were performed using the SPSS statistics V23.0 software (IBM Corporation, Armonk, NY, USA). Significant differences between the two groups were determined using Student’s *t*-test (two-tailed). Meanwhile, differences between more than two groups were determined using a one-way analysis of variance (ANOVA) test followed by Tukey’s (equal variance) or Games-Howell’s (not equal variance) post hoc test. For the Kaplan-Meier curves, *P* values were assessed by the log-rank test. *P* value <0.05 was considered significant.

## 3 Results

### 3.1 *ALDOA* knockout markedly inhibits HCC proliferation both *in vitro* and *in vivo*

**Fig. 1:**
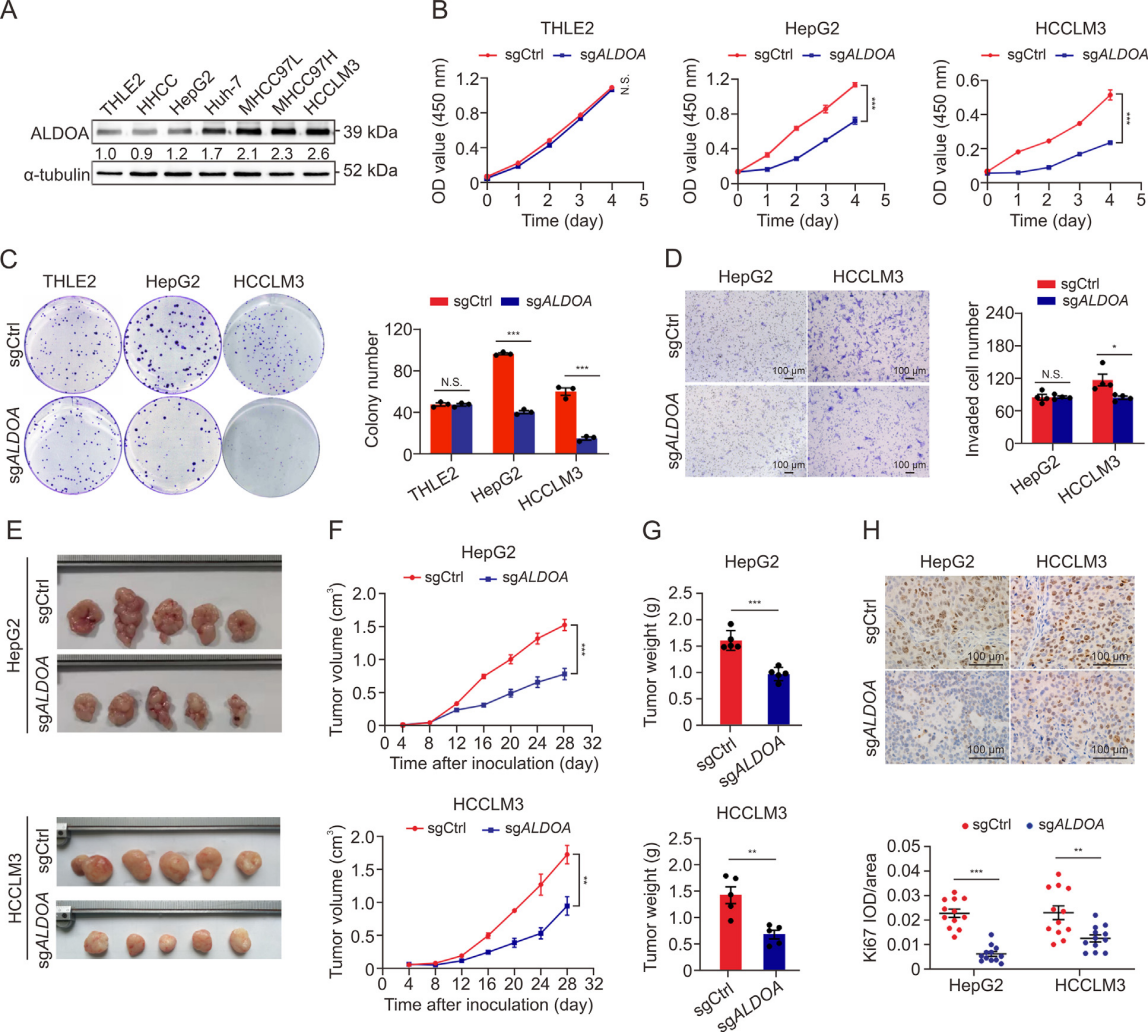
Effect of aldolase A (ALDOA) knockout on tumorigenicity of human hepatocellular carcinoma (HCC) cells both *in vitro* and *in vivo*. (A) The protein expression of ALDOA in six HCC cell lines and the normal human liver cell line, THLE2 (*n* = 3). (B) Cell proliferation curves of THLE2, HepG2, and HCCLM3 cells stably expressing gene knockout of *ALDOA* (sg*ALDOA*) or nontargeting control (sgCtrl) (*n* = 6). (C) Representative images and quantitative analysis of colony formation assay of the above cells (*n* = 3). (D) Representative images and the quantification of transwell matrigel invasion assay (*n* = 4). (E) The images of tumor samples from xenograft mice at 4 weeks after transplantation. (F) The volume of tumors was monitored every 4 days (*n* = 5). (G) Tumor weights of the xenograft mice at 4 weeks after transplantation (*n* = 5). (H) Representative images of Ki67 immunohistochemical staining and quantification (*n* = 12). N.S.: no significant difference, ^∗^*P* < 0.05, ^∗∗^*P* < 0.01, ^∗∗∗^*P* < 0.001. OD: optical density, IOD: integrated optical density.

To explore the biological function of ALDOA in HCC, we detected the protein levels in six HCC cell lines and observed higher levels of ALDOA in Huh-7, MHCC97L, MHCC97H, and HCCLM3 cells, and similar levels in HHCC and HepG2 cells compared to normal liver THLE2 cells (Fig. 1A). Stable gene knockout of *ALDOA* (sg*ALDOA*) and nontargeting control (sgCtrl) using CRISPR and CRISPR-associated protein 9 system were then established in HepG2, HCCLM3, and normal liver THLE2 cells (Figs. S1A and B). *ALDOA* knockout significantly reduced cell proliferation (Figs. 1B and C) and increased the percentage of cells in the G1 phase (Fig. S1C) in HCC cells, but had a weak effect in THLE2 cells. Moreover, *ALDOA* knockout inhibited the 81.8% migration and 59.3% invasion of HCCLM3 cells, while no significantly effect in HepG2 cells Fig. S1D(Figs. 1D and S1D). Collectively, the *in vitro* assay indicated that *ALDOA* could effectively boost HCC cell proliferation. To further verify the effect of *ALDOA* on HCC cell proliferation and tumorigenesis *in vivo*, we performed a subcutaneous xenograft model and an orthotopic xenograft model in nude mice. In the subcutaneous xenograft model, *ALDOA* knockout in HepG2 cells and HCCLM3 cells significantly inhibited tumor size and tumor weight compared to the sgCtrl groups (Figs. 1E–G), similar to the effects *in vitro*. The decreased cell proliferation in *ALDOA* knockout tumors was confirmed by the levels of Ki67, a marker of cell proliferation (Fig. 1H). To further investigate the effect of *ALDOA* on invasiveness, highly aggressive HCCLM3 cells were xenotransplanted orthotopically in the livers of nude mice. Consistently, the tumor volume of the HCCLM3-sg*ALDOA* group was markedly smaller than the sgCtrl group (Fig. S2A), while xenograft tumors of *ALDOA* knockout group displayed similar intrahepatic dissemination and pulmonary metastases to the sgCtrl group on week 6 after transplantation (Figs. S2B–D). These *in vivo* results confirm that *ALDOA* deficiency dramatically suppresses HCC cell proliferation but has a limited impact on tumor infiltration and metastasis.

Conversely, stable overexpression of *ALDOA* was established in HepG2 cells (Fig. S3A). Compared with the controls, HepG2-*ALDOA* cells had greater cell proliferation and a decreased proportion of cells in the G1 phase (Figs. S3B–D), but *ALDOA* overexpression could not produce significant promotion in migratory and invasive capacities (Figs. S3E and F). Furthermore, *in vivo* study showed that *ALDOA* overexpression in HepG2 cells remarkably increased tumor volumes and weights in nude mice (Figs. S3G–J). The increased cell proliferation of *ALDOA* overexpression *in vivo* was further confirmed by histopathological examination and Ki67 levels (Figs. S3K and L). Taken together, these data demonstrate that *ALDOA* is an important facilitator of HCC growth.

### 3.2 ALDOA deficiency partially limits the glycolytic flux in HCC cells

**Fig. 2:**
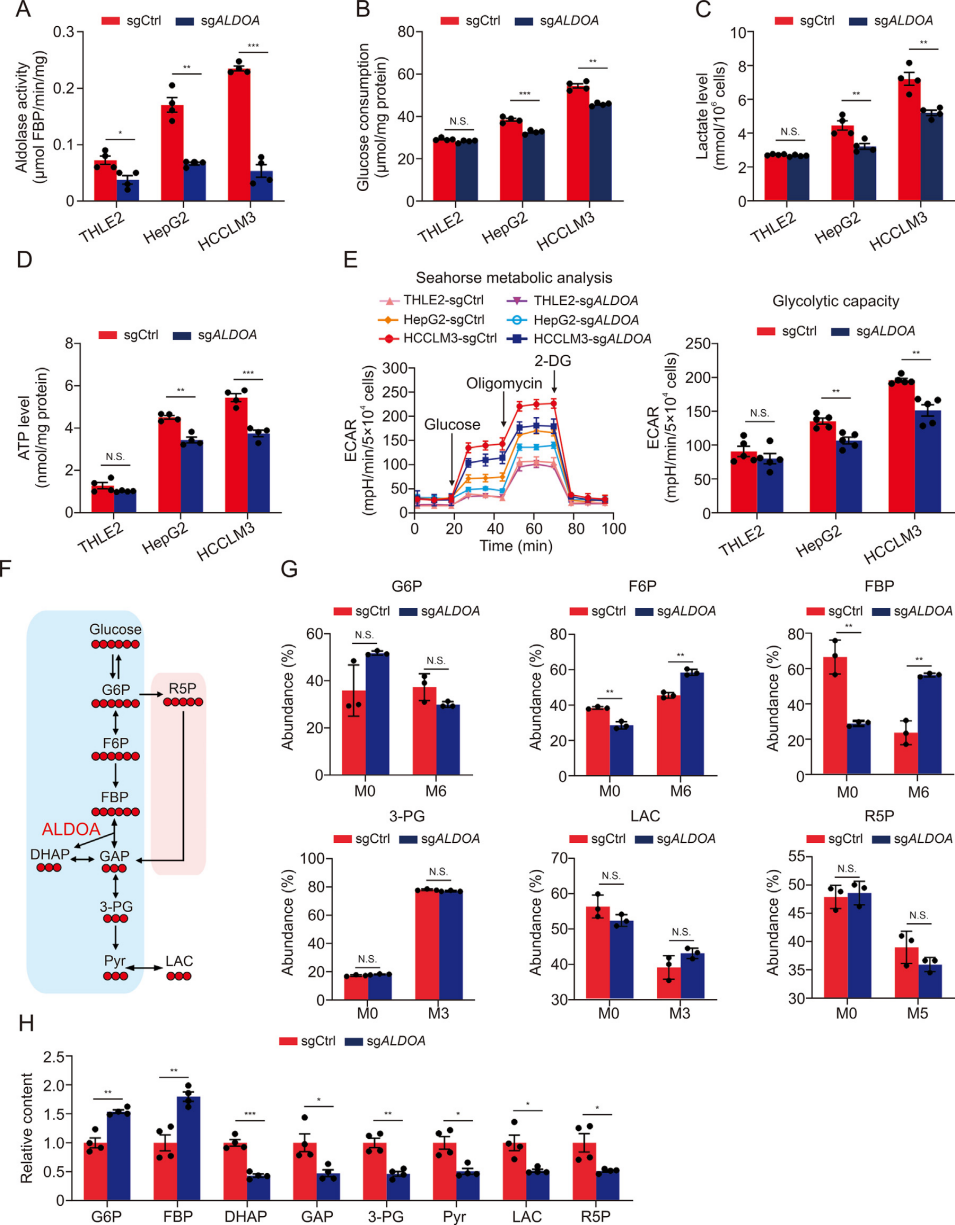
The effect of aldolase A (ALDOA) deficiency on intracellular flux from glucose into lactate. (A–D) The aldolase activity (A), glucose consumption levels (B), lactate secretion levels (C), and adenosine triphosphate (ATP) production levels (D) in three cell lines transfected with gene knockout of *ALDOA* (sg*ALDOA*) or nontargeting control (sgCtrl) (*n* = 4). (E) Seahorse metabolic analysis and calculations of the indicated glycolytic parameters of extracellular acidification rate (ECAR) in three cell lines (*n* = 5). (F) Carbon fate map showing the isotope distribution of indicated metabolites derived from [U–^13^C] glucose. ^13^C atoms are colored in red. (G) Metabolic flux distributions in HCCLM3-sg*ALDOA* and HCCLM3-sgCtrl cells. Fluxes were determined by integrating mass isotopic labeling data from [U–^13^C] glucose tracer experiments at 6 h (*n* = 3). (H) The main metabolic products of glycolysis in HCCLM3-sg*ALDOA* and HCCLM3-sgCtrl cells determined by liquid chromatography-mass spectrometry (LC-MS) (*n* = 4). N.S.: no significant difference, ^∗^*P* < 0.05, ^∗∗^*P* < 0.01, ^∗∗∗^*P* < 0.001. FBP: fructose 1,6-bisphosphate; 2-DG: 2-deoxy-d-glucose; G6P: glucose 6-phosphate; R5P: ribulose 5-phosphate; F6P: fructose 6-phosphate; DHAP: dihydroxyacetone phosphate; GAP: glyceraldehyde 3-phosphate; 3-PG: 3-phosphoglycerate; Pyr: pyruvic acid; LAC: lactate; M: mass isotope distributions.

Since ALDOA is a glycolytic enzyme, whether glucose metabolic change due to ALDOA deficiency impedes HCC progress was further examined in HCC and normal liver cells. As expected, *ALDOA* knockout markedly inhibited the catalytic activity of aldolase enzyme in HCC cells (Fig. 2A), and *ALDOA* knockout significantly decreased glucose consumption, lactate secretion, and ATP production in HCC cells but not in normal liver cells (Figs. 2B–D). Moreover, the ECAR analysis showed that oligomycin stimulation exhibited higher glycolytic capacity in HCC cells than THLE2 cells, and *ALDOA* knockout reduced ECAR levels in HCC cells, suggesting a direct effect of ALDOA on glycolysis (Fig. 2E).

To further assess the effect of *ALDOA* knockout on the glycolytic pathway, we conducted MFA in HCCLM3 cells using [U–^13^C] glucose and [U–^13^C] glutamine as tracers at different time points. ^13^C-labeling analysis of intracellular metabolites converted from [U–^13^C] glucose demonstrated that *ALDOA* knockout increased 1.28-fold ^13^C M6 fructose 6-phosphate (F6P) and 2.38-fold M6 FBP derived from glucose carbons compared to the control group at 6 h (Figs. 2F and G, and Table S2). At 20 h, the fractions of M6 F6P and M6 FBP in *ALDOA* knockout cells were 1.10-fold and 1.32-fold of control cells (Fig. S4 and Table S2). In brief, *ALDOA* knockout led to the accumulation of its upstream metabolites, F6P and FBP, compared to control cells; A more significant accumulation was noted at 6 h compared to 20 h post-intake of ^13^C-glucose. Moreover, the levels of M2 citric acid and M2 succinic acid derived from glucose carbons were reduced to 93.0% and 55.7%, respectively, following *ALDOA* knockout. This finding indicates that *ALDOA* knockout exerts a certain degree of inhibition on the carbon flux entering the tricarboxylic acid (TCA) cycle. To further confirm this inference, MFA data of [U–^13^C] glutamine at 20 h indicated that the relative influxes of glutamine to generate M1 glutamic acid, M1 succinic acid, M1 malic acid, and M1 aspartic acid were partially inhibited by *ALDOA* knockout, indicating that *ALDOA* knockout could appropriately affect the glutaminolysis and several amino acid metabolisms in TCA flux (Figs. S5, and Table S2).

Finally, we measured the intracellular relative abundance of selected glycolytic intermediates in HCCLM3-sg*ALDOA* cells and their control cells during normal cell growth using LC-MS approach. Consistent with the MFA findings, *ALDOA* knockout caused an accumulation of ALDOA upstream metabolites (glucose 6-phosphate and FBP). In comparison, *ALDOA* knockout significantly reduced the levels of its downstream glycolytic intermediates including DHAP, GAP, 3-phosphoglycerate, pyruvic acid, lactate, and ribose-5-phosphate in HCC cells (Fig. 2H). Particularly, the levels of DHAP and GAP, the products from FBP catalyzed by ALDOA, were markedly decreased to 43.3% and 47.5% of control levels in *ALDOA* knockout cells, respectively. Overall, *ALDOA* knockout hampers the splitting of FBP into DHAP and GAP, inhibits subsequent glycolysis, and thereby moderately influences the TCA cycle metabolism.

### 3.3 ALDOA interacts with c-Jun in the nuclei of HCC cells

**Fig. 3:**
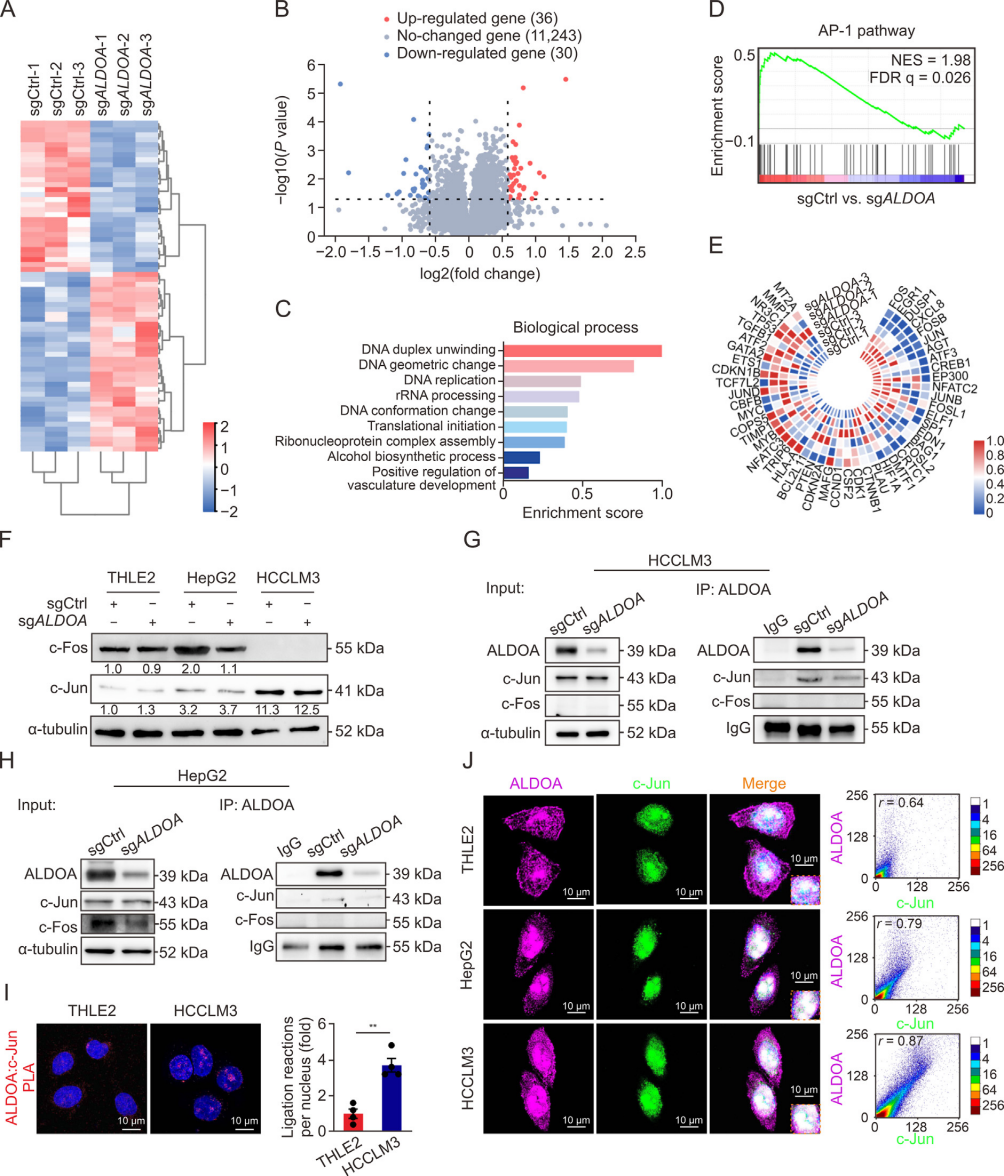
Aldolase A (ALDOA) interacts with c-Jun in the nuclei of hepatocellular carcinoma (HCC) cells. (A) Cluster analysis heat map of differential gene expression in HCCLM3-gene knockout of *ALDOA* (sg*ALDOA*) and HCCLM3-nontargeting control (sgCtrl) cells using RNA sequencing (RNA-Seq). (B) Volcano plot of the changed genes in RNA-Seq between HCCLM3-sg*ALDOA* and sgCtrl cells. (C) Gene Ontology enrichment analysis of the top-ranked genes altered by *ALDOA* in HCCLM3 cells based on the biological process classification of RNA-Seq analysis. (D) The most prominently changed pathway in RNA-Seq evaluated by Gene Set Enrichment analysis. (E) Heat map showing the genes involved in the activator protein-1 (AP-1) pathway in the RNA-Seq analysis. (F) The effects of *ALDOA* knockout on protein expressions of the two subunits of AP-1, c-Jun, and c-Fos, in the whole-cell lysate of three kinds of cells (*n* = 3). (G, H) The co-immunoprecipitation analysis of ALDOA interaction with c-Jun in HCCLM3 (G) and HepG2 (H) cells (*n* = 3). (I) ALDOA-c-Jun interactions in THLE2 and HCCLM3 cells. Representative proximity ligation assay (PLA) photomicrographs (left panel) and the statistical analysis (right panel) are shown (*n* = 4). (J) Immunofluorescence microscopy analysis of ALDOA and c-Jun in three kinds of cells. The 2D correlation scatter diagrams of fluorescence intensity (right panel) show the co-localization of ALDOA and c-Jun in the nuclei (labeled with 4′,6-diamidino-2-phenylindole). ^∗∗^*P* < 0.01. NES: normalized enrichment score; FDR: false discovery rate; IP: immunoprecipitation.

To explore the nonmetabolic effect of ALDOA, we performed an RNA-Seq in HCCLM3-sg*ALDOA* and HCCLM3-sgCtrl cells. Bioinformatic analyses uncovered 66 genes with |fold change| > 0.58 (adjusted *P* < 0.05) in sg*ALDOA* cells compared with sgCtrl (Figs. 3A and B, and Table S3). Gene Ontology analysis in the biological process showed that the top three ranked gene sets were DNA duplex unwinding, DNA geometric change, and DNA replication (Fig. 3C). Gene set enrichment analysis demonstrated that the activator protein-1 (AP-1) pathway was most prominently involved in *ALDOA* knockout cells (Fig. 3D); *FOS*, *EGR1*, *DUSP1*, *CXCL8*, *FOSB*, and *JUN* were the six top-ranked down-regulated genes in the AP-1 pathway (Fig. 3E). The Jun and Fos protein families are essential components of the dimeric AP-1 transcription factor complex. At the protein level, HCC cells exhibited remarkably upregulated c-Jun expression compared with THLE2 cells, and its level in HCCLM3 cells was higher than in HepG2 cells (Fig. 3F). Interestingly, *ALDOA* knockout markedly inhibited c-Fos expression in HCC cells, whereas it had no significant effect on c-Jun expression. In fact, the co-IP assay further showed that ALDOA could directly interact with c-Jun but not c-Fos in HCC cells (Figs. 3G and H). PLA and immunofluorescence analysis were used to confirm the interactions between ALDOA and c-Jun in the nuclei (Figs. 3I, 3J and S6). Compared to normal THLE2 cells, ALDOA and c-Jun interactions were notably higher in HCCLM3 cells. In addition, there was significantly increased colocalization of ALDOA and c-Jun in the nuclei of HepG2 and HCCLM3 cells.

### 3.4 ALDOA facilitates PAK2 to phosphorylate c-Jun at Thr93 site in HCC cells

**Fig. 4:**
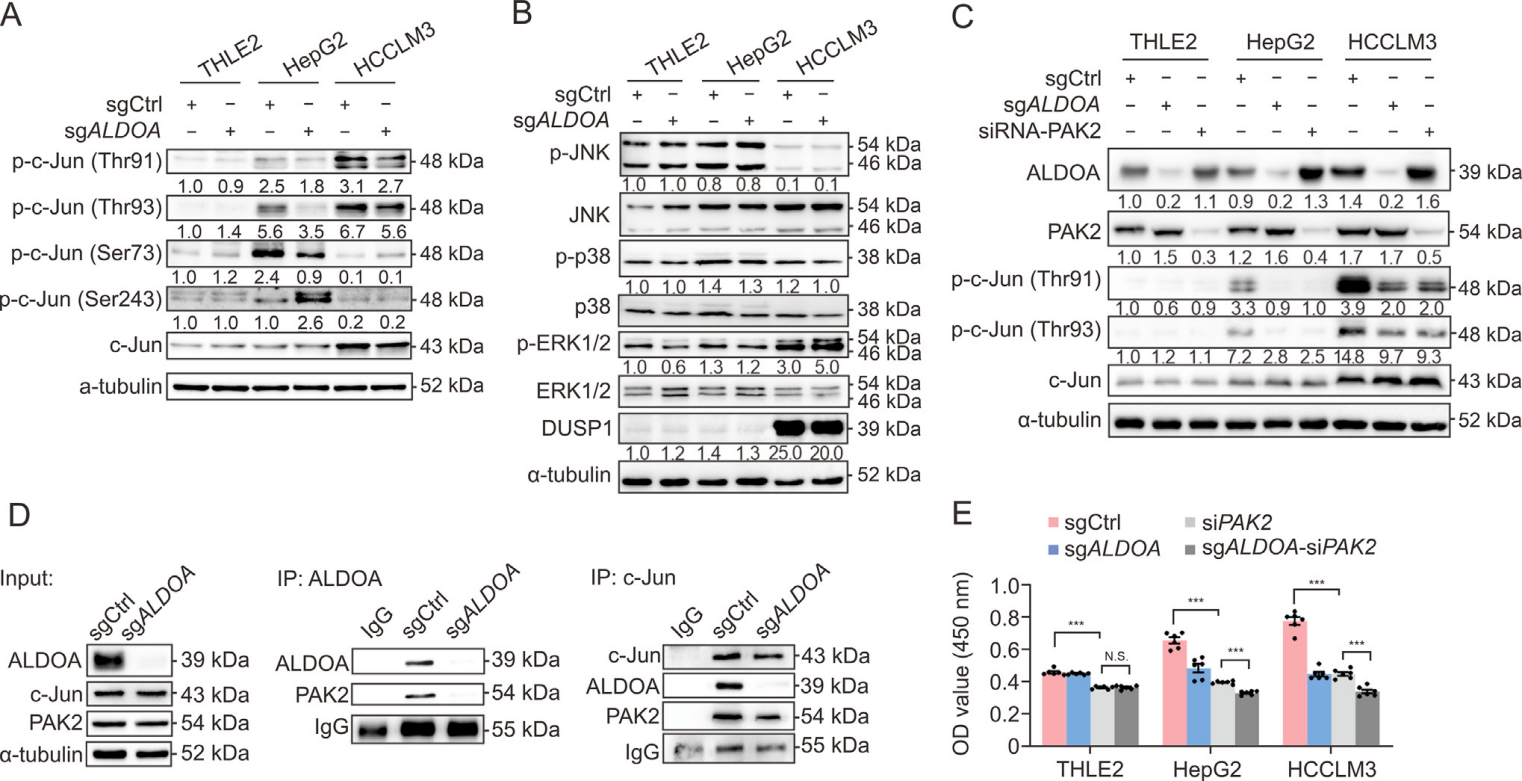
Aldolase A (ALDOA) facilitates c-Jun phosphorylation at the Thr93 site by P21-activated kinase 2 (PAK2) in hepatocellular carcinoma (HCC) cells. (A) The effects of *ALDOA* knockout on phosphorylated c-Jun (p-c-Jun) in three kinds of cells (*n* = 3). (B) The effects of *ALDOA* knockout on mitogen-activated protein kinase (MAPK) activation and DUSP1 expressions in three kinds of cells (*n* = 3). (C) The effects of *PAK2* silencing (si*PAK2*) on the expressions of p-c-Jun and ALDOA (*n* = 3). (D) Co-immunoprecipitation analysis of the interaction among ALDOA, c-Jun, and PAK2 in HCCLM3 cells (*n* = 3). (E) The effects of si*PAK2* on cell proliferation of THLE2, HepG2, and HCCLM3 cells regulated by *ALDOA* (*n* = 6). N.S.: no significant difference, ^∗∗∗^*P* < 0.001. sg*ALDOA*: gene knockout of *ALDOA*, sgCtrl: nontargeting control, p-c-Jun: phosphorylated c-Jun; JNK: c-Jun N-terminal kinases; p-JNK: phosphorylated Jun N-terminal kinases; ERK: extracellular regulated protein kinases; p-ERK: phosphorylated extracellular regulated protein kinases; p-p38: phosphorylated p38; IP: immunoprecipitation; OD: optical density.

The biological function of c-Jun as a transcription factor has been reported to be regulated by three phosphorylation systems. Among them, phosphorylation of the NH_2_-terminally located residues, Ser63, Ser73, Thr91, and Thr93, can enhance the transcriptional activity of c-Jun [23,24], whereas the COOH-terminal cluster of Thr231, Ser243, and Ser249 precludes DNA binding and Ser243 phosphorylation is a priming event for the phosphorylation of the COOH-terminal cluster [25]. Hence, we measured the phosphorylation levels of these key sites in three cell lines and marvelously found that the phosphorylated c-Jun (p-c-Jun) expressions at Thr91 and Thr93 were markedly increased in HCC cells compared with THLE2 cells (Fig. 4A). More importantly, *ALDOA* knockout significantly reduced p-c-Jun at Thr91 and Thr93 sites both in HepG2 and HCCLM3 cells. By contrast, *ALDOA* knockout increased p-c-Jun (Ser243) in HepG2 cells, but not in THLE2 and HCCLM3 cells. As for the p-c-Jun (Ser73) level, it was down-regulated in HepG2 cells but slightly up-regulated in THLE2 cells by *ALDOA* knockout. It is well-established that the activation of mitogen-activated protein kinases (MAPKs), including c-Jun N-terminal kinases (JNK), extracellular regulated protein kinases (ERK), and p38, potentially controls the phosphorylation of c-Jun. Western blotting analysis revealed that *ALDOA* knockout unaffected the phosphorylation of JNK and p38, whereas it distinctly activated ERK in HCCLM3 cells (Fig. 4B). Given that DUSP1 can reciprocally inhibit ERK phosphorylation [26], the knockout of *ALDOA* might increase the activity of ERK via DUSP1 inhibition. These data indicated that MAPKs did not directly participate in the phosphorylation of c-Jun (Thr91 and Thr93) regulated by ALDOA.

It has been reported that PAK2 could phosphorylate c-Jun at Thr93 site, contributing to its transcriptional activation in mouse cells [23]. As expected, *PAK2* silencing significantly diminished the phosphorylation of Thr93 and Thr91 c-Jun in HCC cells, whereas it had no effects on total c-Jun and ALDOA expressions (Fig. 4C). It is worth noting that ALDOA could co-immunoprecipitate with PAK2 in HCC cells using ALDOA as a bait protein (Fig. 4D). Meanwhile, there was an obvious interaction between c-Jun and PAK2. Further cell proliferation assay demonstrated that *PAK2* silencing markedly decreased the cell proliferation compared with control cells (Fig. 4E); *ALDOA* knockout slightly inhibited the proliferation in *PAK2* silencing HCC cells but could not further enhance the inhibitory effect of *PAK2* silencing on THLE2 cells. These findings strongly suggest that the promotion of c-Jun (Thr93) phosphorylation by PAK2 may be a novel mechanism of ALDOA-mediated hepatocarcinogenesis.

### 3.5 The interaction of Y364 ALDOA with Y10 c-Jun is crucial for the phosphorylation of c-Jun at Thr93 site

**Fig. 5:**
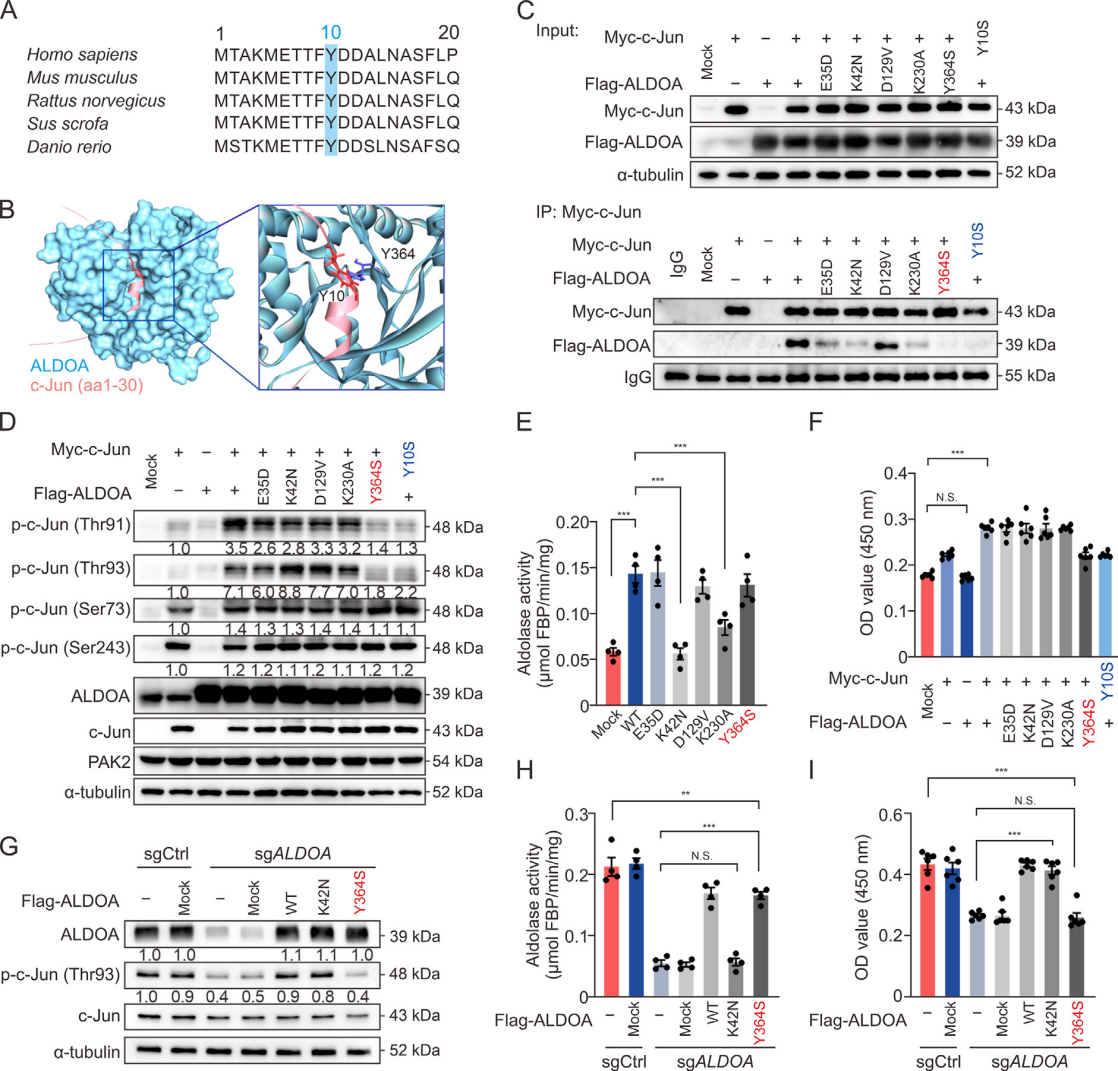
The interaction of Y364 aldolase A (ALDOA) with Y10 c-Jun is crucial for the phosphorylation of c-Jun and hepatocellular carcinoma (HCC) proliferation. (A) Sequence alignment of the conserved tyrosine residues in c-Jun of different species. (B) Schematic diagrams of the N-terminal sequence (aa1–30) of the c-Jun binding pocket in human ALDOA (PDB code: 1ALD) and the interacting residues identified by docking analysis. (C) Co-immunoprecipitation analysis to identify the interaction residues between ALDOA and c-Jun in Flag-ALDOA and/or Myc-c-Jun transiently transfected HEK293T cells (*n* = 3). (D) The effects of different *JUN* or *ALDOA* mutations on the phosphorylation of c-Jun in HEK293T cells after 24 h transfection (*n* = 3). (E) The effects of different *ALDOA* mutations on aldolase activities in HEK293T cells after 48 h transfection (*n* = 4). (F) The effects of different *JUN* or *ALDOA* mutations on the proliferation of HEK293T cells after 48 h transfection (*n* = 6). (G–I) The rescued effects of *ALDOA* mutations on protein expressions of phosphorylated c-Jun (p-c-Jun) Thr93 and ALDOA (G), aldolase activities (H), and cell proliferation (I) in HCCLM3-gene knockout of *ALDOA* (sg*ALDOA*) cells after wild-type (WT) or mutated *ALDOA* transfected for 24 h (protein expression, *n* = 3), 48 h (aldolase activities, *n* = 4), or 72 h (cell proliferation, *n* = 6). N.S.: no significant difference, ^∗∗^*P* < 0.01, ^∗∗∗^*P* < 0.001. PAK2: P21-activated kinase 2; FBP: fructose 1,6-bisphosphate; sgCtrl: nontargeting control; OD: optical density.

One or two tyrosine residues surrounded by acidic amino acids in a short sequence can be the aldolase-binding site [27]. Based on the comparisons of c-Jun’s sequence, Y10 is the possible amino acid interacting with ALDOA, and it is conserved in many species (Fig. 5A). Meanwhile, we performed molecular docking and found that the potential interaction site with Y10 of c-Jun might be Y364 of ALDOA (Fig. 5B). Moreover, it has been reported that E35, K42, and K230 are the ALDOA catalytic sites [28,29], and D129 is pivotal for dimeric ALDOA to form a tetramer [30]. Therefore, we utilized the E35D, K42N, D129V, K230A, and Y364S mutants of ALDOA, as well as the Y10S c-Jun mutant, to determine which regions of ALDOA interact with c-Jun. The co-IP experiments revealed that wild-type (WT) c-Jun interacted with ALDOA, but the Y10S mutant c-Jun damped their interaction (Fig. 5C). Furthermore, c-Jun could not bind to the Y364S mutant, but could bind to exogenously expressed WT, E35D, K42N, D129V, and K230A ALDOA. These findings indicate that the Y364 of ALDOA and the Y10 of c-Jun are the pivotal sites for their interaction. Based on the key interaction sites identified above, we further explored the possibility that the activation of c-Jun might be caused by its binding with ALDOA. We found that Thr91/Thr93 c-Jun phosphorylation levels were quite low among the four phosphorylated sites in solely c-Jun-expressing cells (Fig. 5D). In addition, co-expression of ALDOA and c-Jun strikingly increased Thr91/Thr93 c-Jun phosphorylation levels. Consistent with previous results, only the Y364S mutant displayed no significant phosphorylation of c-Jun at Thr91/Thr93 among all ALDOA mutations; the Y10S mutant of c-Jun demonstrated analogous effects. Collectively, these results confirm, alongside the docking analysis, that Y364 ALDOA and Y10 c-Jun are the main protein interaction sites, and their interaction is crucial for the phosphorylation of c-Jun at Thr91/Thr93 by PAK2.

### 3.6 The interaction of ALDOA with c-Jun is responsible for malignancy proliferation in HCCs

To elucidate the effects of ALDOA-c-Jun interaction on enzyme activity and cell proliferation, WT or mutant ALDOA and c-Jun co-expressed cells were further analyzed. Apart from the K42N and K230A mutations, all other mutations demonstrated significant aldolase activity (Fig. 5E), further confirming that K42 and K230 were the catalytic sites of ALDOA with substrate FBP. Moreover, the co-expression of ALDOA and c-Jun significantly increased cell proliferation (Fig. 5F). All ALDOA mutants significantly enhanced cell proliferation, with the exception of the Y364S mutation, which exhibited a slight increase in cell proliferation at a level comparable to that of Y10S c-Jun. The importance of the interaction between ALDOA and c-Jun in regulating cell proliferation was further validated by the rescue experiment. HCCLM3-sg*ALDOA* cells were expressed with Y364S, K42N, or WT ALDOA. We found that impaired c-Jun Thr93 phosphorylation, enzymatic activity, and cell proliferation after *ALDOA* knockout could be dramatically restored by the expression of WT ALDOA (Figs. 5G–I). It is worth noting that c-Jun phosphorylation at Thr93 and cell proliferation were markedly regained in the cells transfected with K42N ALDOA, almost to WT levels, despite that these cells could not recover enzymatic activity. However, the Y364S mutant restored enzymatic activity comparable to that of the WT ALDOA, yet it failed to recover c-Jun phosphorylation or cell proliferation. Hence, ALDOA binding to c-Jun is essential for promoting HCC proliferation, which is principally enzymatic activity-independent.

### 3.7 ALDOA enhances c-Jun transcriptional activity to increase *ALDOA* autologous transcription in HCCs

**Fig. 6:**
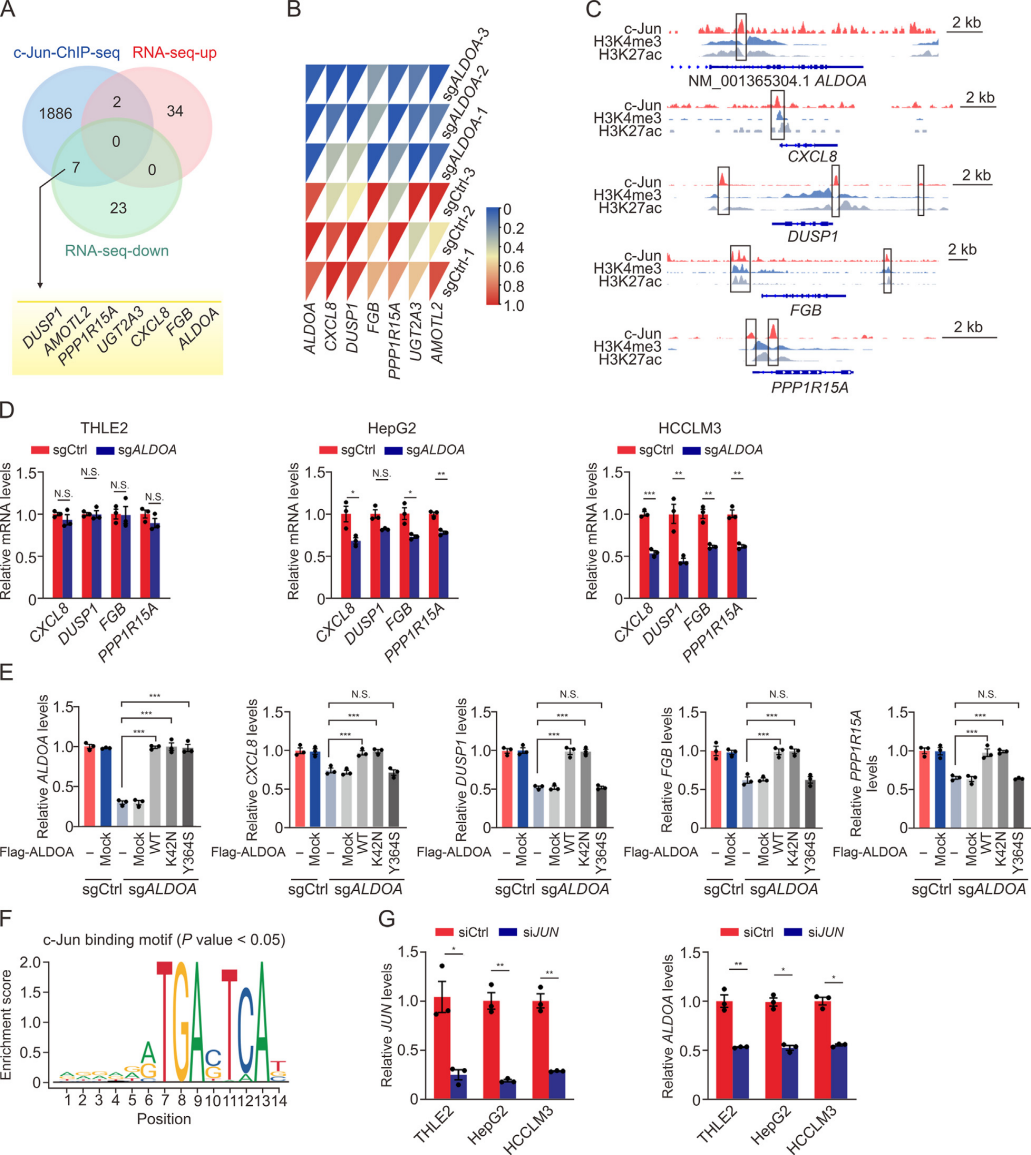
Transcriptional targets of c-Jun regulated by aldolase A (ALDOA) in hepatocellular carcinoma (HCC) cells. (A) Venn diagram depicting the transcriptional regulation of c-Jun in chromatin immunoprecipitation sequencing (ChIP-Seq) data (GSM935364) of HepG2 cells and the predicted target genes of c-Jun regulated by ALDOA in HCCLM3 cells. The bottom panel shows the predicted target genes. (B) Heat map showing the predicted target genes of c-Jun activation regulated by ALDOA in HCCLM3 cells using RNA sequencing (RNA-seq). (C) c-Jun occupancy at *ALDOA*, *CXCL8*, *DUSP1*, *FGB*, and *PPP1R15A* gene loci in ChIP-Seq data (GSM935364) of HepG2 cells. (D) The messenger RNA (mRNA) levels of *CXCL8*, *DUSP1*, *FGB*, and *PPP1R15A* in THLE2-gene knockout of *ALDOA* (sg*ALDOA*), HepG2-sg*ALDOA*, HCCLM3-sg*ALDOA* cells, and their control cells (*n* = 3). (E) The rescued effects of *ALDOA* mutations on the levels of c-Jun target genes in HCCLM3-sg*ALDOA* cells after transfection for 48 h (*n* = 3). (F) The predicted c-Jun-binding sites at the promoters of *ALDOA*. (G) The effects of *JUN* silencing (si*JUN*) on the mRNA expressions of *ALDOA* in three kinds of cells. The mRNA levels of *JUN* and *ALDOA* were measured by quantitative real-time PCR (qPCR) assay in the cells treated with *JUN* small interfering RNA (siRNA) for 48 h (*n* = 3). N.S.: no significant difference, ^∗^*P* < 0.05, ^∗∗^*P* < 0.01, ^∗∗∗^*P* < 0.001. sgCtrl: nontargeting control.

c-Jun is a potent transcriptional activator that positively regulates the carcinogenesis process. Although much has been discovered about the transcriptional regulation of c-Jun phosphorylation at Ser63/73, its phosphorylation of Thr93 ensuing transcriptional activation remains poorly understood [23]. Because ALDOA enhances the phosphorylation of c-Jun at Thr93, we further determined whether and how the presence of ALDOA affected the binding of c-Jun to its transcriptional targets. A ChIP-Seq profiles (GSM935364) of HepG2 was obtained for peak analysis; there were 1,895 gene promoters of transcription start sites binding to c-Jun using the ChIPseeker tool analysis (Fig. 6A). By comparing changes in these binding genes in our RNA-Seq analysis, there were two significantly upregulated and seven downregulated genes. We further selected the five top-ranked downregulated genes, *ALDOA, CXCL8, DUSP1*, *FGB*, and *PPP1R15A*, to validate the analysis results (Fig. 6B) and found that c-Jun could directly bind to the genomic regions of these genes (Fig. 6C). Among these genes, *CXCL8* and *PPP1R15A*, which participate in pro-tumorigenic cytokine production [31,32], were prominently reduced both in HepG2-sg*ALDOA* and in HCCLM3-sg*ALDOA* cells (Fig. 6D), but not THLE2-sg*ALDOA* cells, indicating that ALDOA regulated the transcription of these c-Jun target genes. To further prove that ALDOA directly affects c-Jun transcriptional activity, we tested these c-Jun target genes in HCCLM3-sg*ALDOA* cells exogenously expressed with WT, Y364S, or K42N ALDOA. As shown in (Fig. 6E), the replenishment of WT and K42N mutant ALDOA drastically increased the levels of all tested c-Jun targets, whereas the Y364S ALDOA supplement could not restore the mRNA levels of these genes.

Interestingly, we predicted that c-Jun could also directly bind to the genomic regions AGGAGATGACTCAT of *ALDOA* (Fig. 6F). This positive feedback regulation could be the reason for the high expression of ALDOA in HCC cells. Hence, *ALDOA* mRNA levels were further measured in c-Jun-silenced three kinds of cells. As shown in (Fig. 6G), *JUN* knockdown could markedly decrease *ALDOA* expressions. These findings depict a map of ALDOA regulating transcriptional activation of c-Jun target genes. Meanwhile, the results present a loop of ALDOA governing its own function.

### 3.8 The high level of ALDOA in HCC patients correlates with p-c-Jun (Thr93) and indicates poor prognosis

**Fig. 7:**
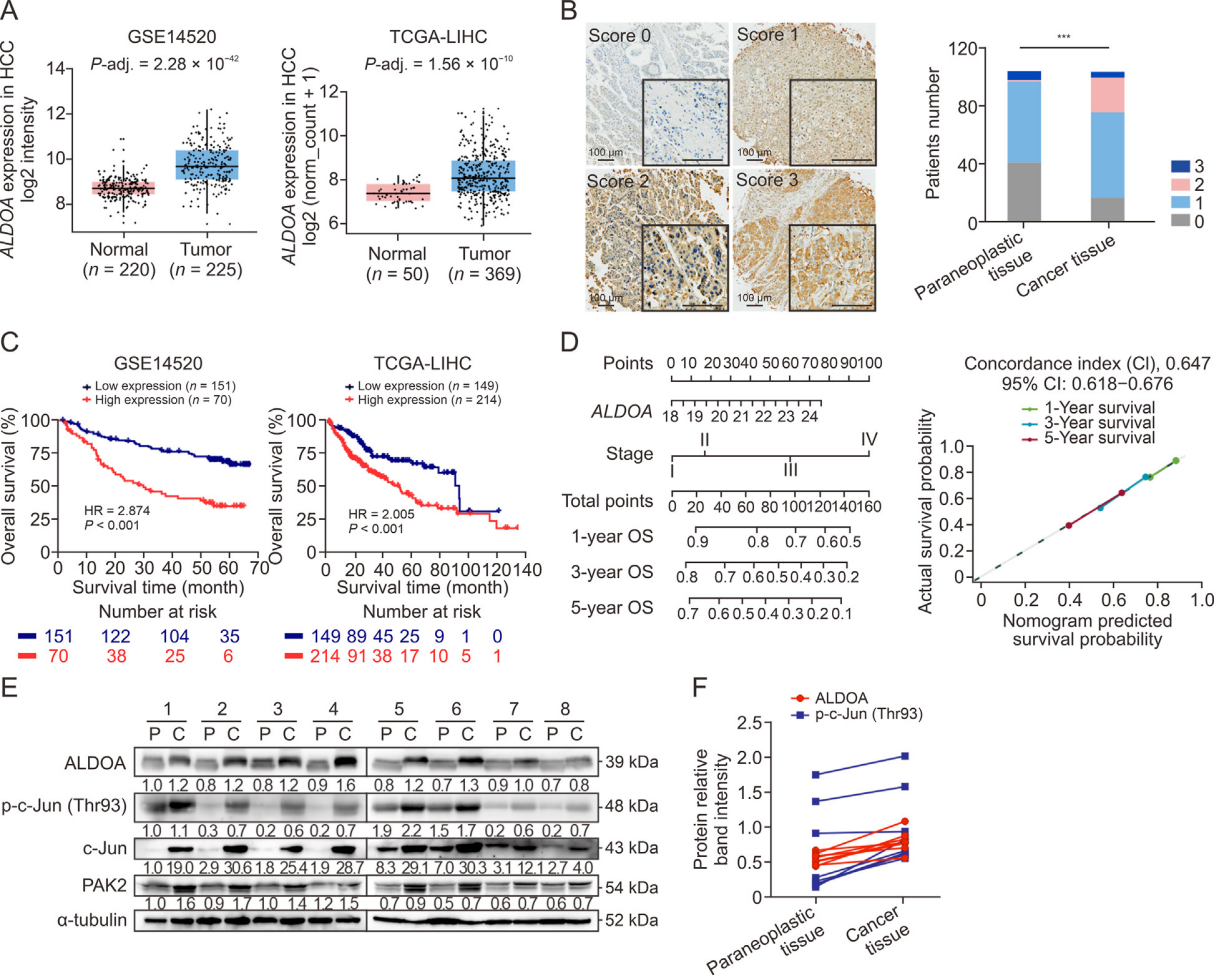
The upregulation of aldolase A (ALDOA) correlated with human hepatocellular carcinoma (HCC) development. (A) Box plots of *ALDOA* messenger RNA (mRNA) expressions in HCC and normal liver tissues from the GSE14520 in Gene Expression Omnibus (GEO) (*n* = 445) and the Cancer Genome Atlas Liver Hepatocellular Carcinoma (TCGA-LIHC) (*n* = 419) datasets, respectively. (B) The scores of ALDOA protein levels in representative tumor tissues from a tissue microarray of 104 paired human HCC specimens. (C) Kaplan-Meier curves of survival differences in *ALDOA*-high and -low expressed HCC patients from the GSE14520 in GEO (221 patients) and TCGA-LIHC (363 patients) datasets (*n* = 584), respectively. Survival difference was evaluated using the Log-rank test. (D) Postoperative prognostic nomogram for patients with HCC, and the calibration curve of the nomogram for predicting the overall survival at 1 year (green), 3 years (blue), and 5 years (red). Actual overall survival (OS) is plotted on the y-axis; nomogram predicted probability of OS is plotted on the x-axis. (E) The protein levels of ALDOA, phosphorylated c-Jun (p-c-Jun) Thr93, c-Jun, and P21-activated kinase 2 (PAK2) in the paraneoplastic (P) and cancer (C) tissues of HCC patients (*n* = 8). (F) Protein expressions of ALDOA and p-c-Jun (Thr93) in 8 paired HCC tissues and adjacent normal tissues. ^∗∗∗^*P* < 0.001. HR: hazard ratio.

To discover the clinical significance of ALDOA, we analyzed public GEO and TCGA-LIHC databases. Increased *ALDOA* and *PAK2* mRNA levels were observed in HCC tissues compared with normal liver tissues, whereas there was no significant correlation between *ALDOA* and *PAK2* (Figs. 7A, S7A and S7B). Immunohistochemical staining analysis confirmed that ALDOA protein levels were upregulated in 104 paired liver tumor specimens and the intensity of ALDOA in the nuclei of tumor tissues dramatically increased (Fig. 7B). The diagnostic value of *ALDOA* upregulation was confirmed by ROC curves in the GEO and TCGA-LIHC databases (*P* < 0.001) (Fig. S7C). According to Kaplan-Meier survival curves, HCC patients with high *ALDOA* expression were significantly associated with poorer overall survival (OS) in these datasets (Fig. 7C). Univariate and multivariate Cox proportional hazard regression analysis revealed that high *ALDOA* expression was an independent risk factor for the poor prognosis of patients with HCC (Tables S4–S7). Considering the heterogeneity of HCC patients, subgroup analysis was performed based on tumor-node-metastasis (TNM) stage and demonstrated that *ALDOA* overexpression was consistently associated with poor survival regardless of TNM stage (Fig. S7D). Finally, we constructed prognostic nomograms based on *ALDOA* mRNA expression and TNM stage, and the calibration plot showed that the nomogram prediction was in excellent agreement with the probability of HCC patient survival (1-, 3-, and 5-years of OS), confirming that *ALDOA* expression could predict the clinical outcome of HCC patients (Fig. 7D).

To evaluate the clinical relevance of ALDOA-mediated c-Jun Thr93 phosphorylation in HCC patients, Western blotting was performed on eight human HCC tissues (Fig. 7E). Consistent with the observations of database analysis and IHC staining, ALDOA protein was expressed at higher level in HCC tissues compared to the adjacent tissues. Interestingly, the levels of c-Jun, p-c-Jun (Thr93), and PAK2 were also markedly increased in HCC tissues, meanwhile there are similar changes between ALDOA and p-c-Jun (Thr93) protein expressions (Fig. 7F). Taken together, these results suggest that the progression of HCC necessitates high levels of ALDOA-mediated c-Jun-Thr93 phosphorylation.

### 3.9 AAV8-medicated *Aldoa* deficiency markedly inhibits tumor progress in DEN-induced HCC mouse models

The enhancing effect of ALDOA on liver tumorigenesis prompted us to evaluate the therapeutic potential of its knockout in HCC. We extended our observations *in vivo* using the well-established DEN model, treated as the schedule illustration (Fig. S8A). As shown in (Figs. S8B and C), the expressions of ALDOA and p-c-Jun (Thr93) were gradually increased in DEN-induced livers. Meanwhile, ALDOA nuclear-positive cells appeared in liver tumor tissues, mainly after 6 months of DEN-treated, by the time of tumor development in the promotion and progression stages [33]. Immunofluorescence co-localization analysis identified gradually increased ALDOA and c-Jun nuclear-positive cells in liver tumor tissues with the time extension of DEN-induction (Fig. S8D). These data indicated that ALDOA was significantly upregulated in the promotion and progression stages, which could play an essential regulatory role in DEN-induced HCC mice.

**Fig. 8:**
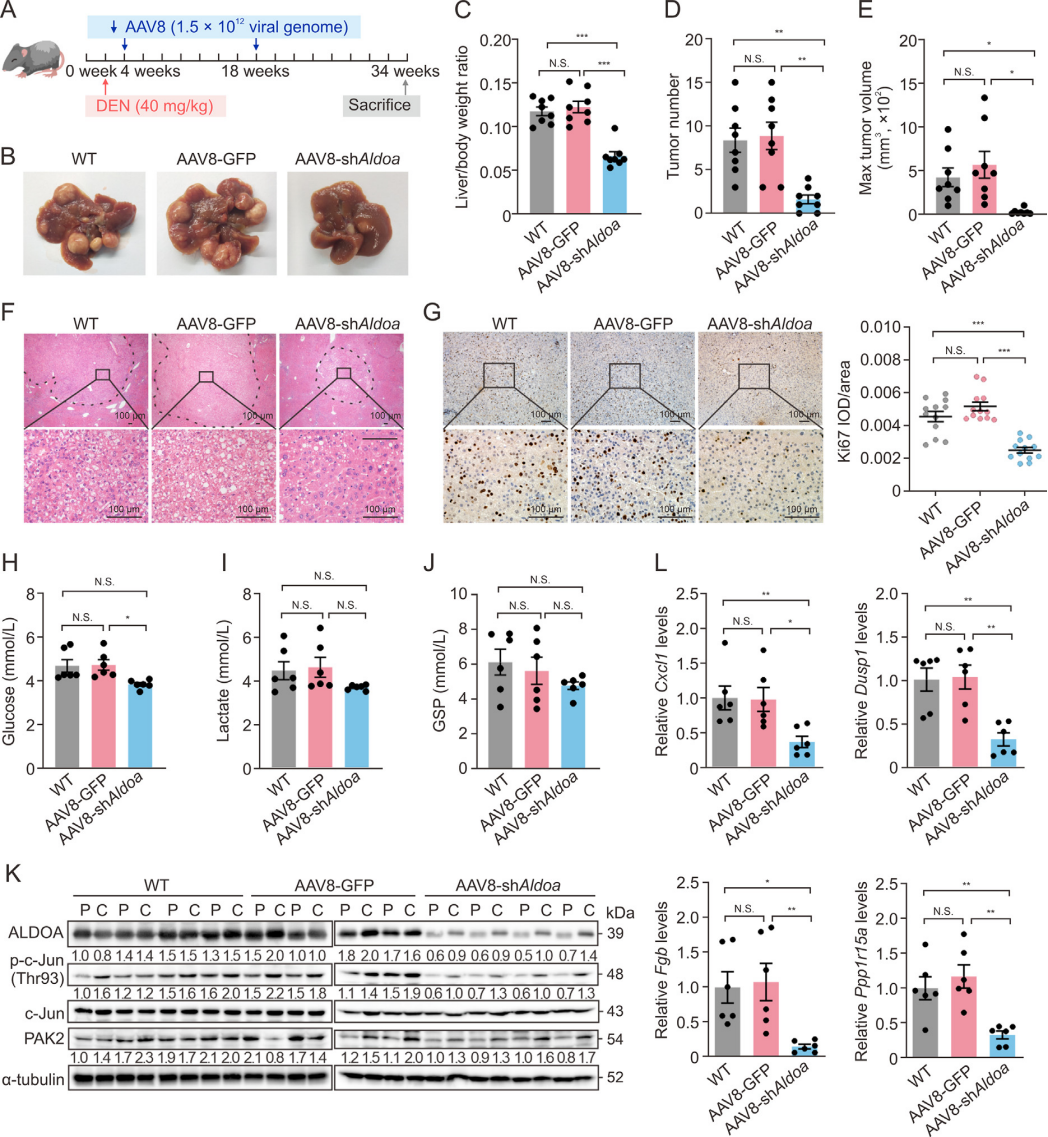
Adeno-associated virus based on serotype 8 (AAV8)-medicated *Aldoa* deficiency decreased the susceptibility of mice to diethylnitrosamine (DEN)-induced hepatocellular carcinoma (HCC). (A) Schematic treatment of DEN challenge (intraperitoneally) and AAV8 intervention (intravenously) on male C57BL/6 mice. (B) Representative images of livers at 8 months after DEN treatment. (C) Quantification of the liver-to-body weight ratios (*n* = 8). (D) The number of macroscopic tumors (≥0.1 cm) identified in animals at the time of sacrifice (*n* = 8). (E) The mean max tumor volumes in three groups (*n* = 8). (F) Representative images of hematoxylin and eosin (H&E) staining in tumor foci and adjacent normal liver. Some tumors in the livers are shown by dotted circles. (G) Representative images of Ki67 immunohistochemistry staining and quantification in tumor foci and adjacent normal liver (*n* = 12). (H–J) The levels of glucose (H), lactate (I), and glycosylated serum protein (GSP) (J) in the serum of DEN-induced mice (*n* = 6). (K) The protein levels of aldolase A (ALDOA), phosphorylated c-Jun (p-c-Jun) Thr93, c-Jun, and P21-activated kinase 2 (PAK2) in the livers of DEN-treated mice (*n* = 4). (L) The messenger RNA (mRNA) levels of *Cxcl1, Dusp1, Fgb,* and *Ppp1r15a* in the livers of DEN-treated mice (*n* = 6). N.S.: no significant difference, ^∗^*P* < 0.05, ^∗∗^*P* < 0.01, ^∗∗∗^*P* < 0.001. WT: wild-type, AAV8-sh*Aldoa*: AAV8 liver-specific *Aldoa* knockdown; AAV8-GFP: AAV8 liver-specific GFP control; IOD: integrated optical density.

Afterward, we exploited AAV8 liver-specific *Aldoa* knockdown (AAV8-sh*Aldoa*) mice, induced with DEN to test the role of *Aldoa* in the development of HCC (Figs. 8A, 8B, and S9A) and evaluate its therapeutic potential. *Aldoa* knockdown in livers was validated by decreases in mRNA level at 8 months, while AAV8-sh*Aldoa* had no impact on its expression in the skeletal muscle and hearts (Fig. S9B). Notably, the liver/body weight ratio, tumor number, and maximum tumor diameter in AAV8-sh*Aldoa* mice were significantly reduced compared to WT or control AAV8 liver-specific GFP control *(*AAV8-GFP*)* mice (Figs. 8C–E, and S9C and D), indicating that *Aldoa* knockdown markedly attenuates tumor formation. Consistent with tumorigenesis, liver cell proliferation and the levels of ALT and AST were significantly decreased in AAV8-sh*Aldoa* mice (Figs. 8F, 8G, and S9E). The serum levels of glucose and lactate respectively decreased to 81.1% and 80.0% by *Aldoa* knockdown (Figs. 8H and I), though no significant difference in GSP among AAV8-sh*Aldoa*, WT, and AAV8-GFP mice was found (Fig. 8J). Consistent with the observations in the orthotopic xenograft model, similar ratios of lung metastases (6/8 vs. 8/8) were found in AAV8-sh*Aldoa* mice compared to AAV8-GFP mice (Fig. S9F), which implies that *Aldoa* knockdown had no noticeable effect on HCC metastasis. Moreover, the expressions of key proteins demonstrated that *Aldoa* knockdown markedly inhibited the expressions of ALDOA and p-c-Jun (Thr93) both in liver cancers and in paracancerous tissues (Fig. 8K). Consistent with the observations *in vitro*, the mRNA levels of c-Jun target genes, *Cxcl1*, *Dusp1*, *Fgb*, and *Ppp1r15a*, regulated by ALDOA were significantly decreased in the cancer tissues of AAV8-sh*Aldoa* mice compared with AAV8-GFP mice (Fig. 8L). In summary, these data further indicate that targeting the ALDOA-p-c-Jun (Thr93) axis is a potential therapeutic strategy for HCC treatment.

## 4 Discussion

HCC ranks as the sixth most frequently diagnosed cancer and the third foremost contributor to cancer-related mortality worldwide [34]. Extensive research efforts have been dedicated to the pursuit of efficacious therapeutic approaches for HCC in recent decades. Particularly, elucidating the canonical and noncanonical functions of metabolic enzymes in cancer cells holds significant promise for the advancement of novel anti-HCC treatments [9]. This study provides evidence that ALDOA plays a role in promoting HCC cell proliferation, and its deficiency can partially inhibit glycolysis and the TCA cycle in HCC. Importantly, a novel moonlighting function of ALDOA plays a major role in regulating HCC proliferation. ALDOA acts as a cofactor of c-Jun, enhancing the transcription of oncogenes in a PAK2-dependent manner after its specific nuclear translocation in HCC cells. Knockdown of *Aldoa* in the liver using AAV8-mediated delivery significantly inhibits the progression of HCC *in vivo*. Overall, our findings reveal that ALDOA regulates proliferation of HCC through glycolysis and c-Jun-mediated oncogene transcription, suggesting that targeting ALDOA could be a potential avenue for anti-HCC therapy.

The current investigation revealed a significant upregulation of ALDOA in numerous cancer types, accompanied by a notable suppression of cancer cell proliferation, migration, and invasion upon *ALDOA* knockdown. Specifically, this study demonstrated that excessive *ALDOA* expression facilitated HCC proliferation and G1/S transition, whereas *ALDOA* knockout effectively curbed HCC proliferation both *in vitro* and *in vivo*, aligning with similar observations in non-small cell lung cancer [11]. Previous studies have reported that knockdown of *ALDOA* inhibited growth and migration of HCC cells under hypoxia [15]. Consistently, our study identified *ALDOA* knockout markedly inhibit the proliferation of HCC cells in normoxia. However, differing from the results in hypoxia, *ALDOA* knockout modestly suppresses the invasion and migration of HCCLM3 cells, yet does not affect those of HepG2 cells. Indeed, it has been reported that the direct evidence of hypoxia in human HCC is sparse [35], as this tumor is hypervascular and receives enough oxygen from the portal vein [17]. Meanwhile, HIF-1α regulates multiple glycolytic enzymes in hypoxic conditions, contributing to the aggressive phenotype of HCC [36]. Therefore, ALDOA may facilitate HCC metastasis in hypoxic conditions while promoting cell survival and proliferation in normoxia. Furthermore, given the considerable heterogeneity of HCC, it is essential to explore the roles of ALDOA under both normoxic and hypoxic conditions.

Metabolic deregulation, a hallmark of malignancy, is mediated by proto-oncogene overactivation, ultimately resulting in the dysregulated production of glycolytic enzymes in tumors [6]. As a classical glycolytic enzyme, it is widely acknowledged that ALDOA can enhance glycolysis in cells [15,37]. Consistently, HCC cells exhibited a noticeable increase in glucose consumption, lactate secretion, ATP production, and the net ECAR compared to normal liver cells. Furthermore, all of these parameters significantly decreased following the *ALDOA* knockout in HCC cells. Here, we used [U–^13^C] glucose to trace the catabolism of glucose in glycolysis. The MFA analysis demonstrated that the knockout of *ALDOA* led to a decrease in glycolytic flux, characterized by the accumulation of F6P and FBP. Meanwhile, *ALDOA* knockout reduced the metabolic fluxes of glutamic acid, succinic acid, malic acid, and aspartic acid. The MFA analysis revealed a transient and singular glucose metabolic process. In order to capture the comprehensive alterations in glycolysis pathway metabolites following *ALDOA* knockout in HCC cells, we conducted further measurements of the overall product levels of glycolysis. These measurements revealed that *ALDOA* knockout increased glucose 6-phosphate and FBP levels while decreasing DHAP, GAP, and lactate, the end product of glycolysis. The presence of lactic acid or an acidic environment is responsible for the invasion and metastasis of cancer cells [38]. Although the knockout of *ALDOA* resulted in a decrease in ECAR and lactate production, this reduction was not sufficient to significantly impede the invasion and migration of HCC cells. It has been reported that the effect of aldolase knockdown on oncogene-transformed cell proliferation is independent of its glycolytic function [39]. The prior research is consistent with our finding [40], that the transfection of catalytically inactive *ALDOA* mutants in *ALDOA*-deficient HCC cells can still notably enhance cell growth (Fig. 5I). Consequently, the excessive expression of ALDOA in HCC may be linked to noncatalytic mechanisms that facilitate cancer progression.

In the past decade, a growing number of studies have reported that the noncanonical functions of metabolic enzymes play crucial roles in tumorigenesis [9]. It has been indicated that ALDOA is classified as a moonlighting enzyme, meaning it participates in cellular processes beyond glycolysis. One specific mechanism that has been extensively studied is the interaction between ALDOA and F-actin [41]. Recent studies have shown that ALDOA can hinder the polymerization of actin through the wiskott-aldrich syndrome protein pathway, thereby enhancing the motility of cancer cells [42]. In addition, several studies have demonstrated that ALDOA promotes tumorigenesis by affecting oncogenic signaling pathways [43], cell cycle [11], and mRNA translation [40]. Interestingly, ALDOA has been found in the nuclear localization of several kinds of cancer cells. Although it has been reported that the nuclear localization of ALDOA correlates with the proliferative activity [44], the specific mechanism of its action is still unclear. We herein observed significant ALDOA nuclear transport in HCC cells and subsequently found the interaction between ALDOA and c-Jun by combining gene expression and molecular biology experiments. The transcription factor c-Jun/AP-1 plays a crucial role in HCC development [34]. The phosphorylation of c-Jun modulates its activities, including its transactivating potential, DNA-binding capacity, and dimer stability. Thus far, it has been established that the phosphorylation of human c-Jun primarily occurs *in vivo* through the action of three specific protein kinases, namely JNK, glycogen synthase kinase-3, and protein kinase 2 [45]. The latter two kinases target the regions adjacent to the C-terminal domain of c-Jun, whereas JNK phosphorylate two distinct groups of sites, namely Ser63&73 and Thr91&93 [46]. The present study showed that *ALDOA* knockout dramatically suppressed p-c-Jun (Thr93) level, but not Ser63&73, which implies that c-Jun activation by ALDOA is not associated with JNK. Previous findings have revealed that PAK2 phosphorylates c-Jun at five threonine sites (Thr2, Thr8, Thr89, Thr93, and Thr286), and exerts a crucial role in epidermal growth factor-induced proliferation and transformation of mouse cell by influencing AP-1 activity [23]. c-Jun is conserved between mouse and human. Meanwhile, a significant correlation has been found between the expression of phosphorylated PAK2 and the progression and metastasis of HCC [47]. Based on this, we hypothesized and subsequently confirmed that PAK2 phosphorylates Thr93 c-Jun, and this process is notably facilitated by ALDOA in human HCC. This suggests that the physical interaction between ALDOA and c-Jun induces a conformational change that increases the accessibility of kinase docking sites for PAK2 protein.

The ALDOA tetramer is composed of a pocket domain and four symmetrical side chains, accompanied by a flexible catalytic C-tail region [41]. Our experimental findings, through site mutations, have revealed that K42 and K230 of ALDOA, with a particular emphasis on K42, play a crucial role in regulating the enzyme activity of ALDOA. However, it is noteworthy that the mutation of these sites did not impede cell proliferation, indicating that the catalytic activity of ALDOA operates independently from its regulatory function in cell proliferation. The molecular docking and site mutations analysis revealed that Y364 of ALDOA is a distinct site involved in the interaction with c-Jun. Furthermore, the suppressed cell proliferation observed in *ALDOA* knockout cells could be restored to nearly normal levels by both WT and K42N mutant ALDOA, but not by the Y364S mutant ALDOA. Although the possibility of other mechanisms contributing to the inhibitory effect of *ALDOA* knockout on cell proliferation cannot be completely ruled out, the observation that K42N ALDOA reinstates proliferation to a level similar to that of the WT suggests that ALDOA primarily promotes cell proliferation through the activation of c-Jun rather than its enzymatic activity.

Except for a case of ALDOA down-regulation in Japanese HCC patients' specimen [4], ALDOA has been reported to be markedly elevated in most HCC specimens. Consistent with these studies [48], our results demonstrated that the expressions of both ALDOA gene and protein levels were upregulated in HCC patients and that high ALDOA expression was an independent risk factor for the poor prognosis of HCC, indicating that ALDOA represents a potential marker for pathologic classification and prognostic evaluation in HCC. Upon hypoxia, the liver gradually develops adaptive responses by activating the HIF-1α-inducible genes involved in glycolysis, such as *ENO1*, *PKM*, *GPI*, and *ALDOA*, through activating transcription [36]. In addition to environmental hypoxia, the overexpression of *ALDOA* can be induced by aberrant oncogenic c-Jun in this study. The utilization of Chip-Seq and PCR analysis revealed that c-Jun is capable of interacting with the *ALDOA* motif and promoting its expression. Conversely, the suppression of *JUN* significantly hindered the expression of *ALDOA*. This study further corroborated that the expressions of ALDOA and Thr93 c-Jun phosphorylation in the tumor tissues were dramatically higher than in adjacent tissues, and ALDOA level was positively associated with Thr93 c-Jun phosphorylation in DEN-induced mice and HCC patients. Compared with c-Jun inhibition, targeting ALDOA could allow for the selective resistance of HCC with a significantly reduced potential for side effects because ALDOA is highly expressed in HCC but lowly in normal hepatocytes. By employing AAV8-mediated liver gene knockdown, the inhibition of DEN-induced HCC formation was notably observed upon *Aldoa* knockdown *in vivo*, thereby indicating the potential of ALDOA inhibition as a viable strategy for HCC therapy. Nevertheless, further investigation is required to ascertain the feasibility of developing small molecule inhibitors specifically targeting ALDOA.

## 5 Conclusion

In conclusion, this study reveals the regulatory role of ALDOA in glycolysis through its canonical enzyme function, as well as a previously unknown moonlighting function in HCC proliferation. The high expression of ALDOA is associated with poor prognosis in HCC patients. *ALDOA* knockout significantly inhibits the proliferation of HCC cells *in vitro* and *in vivo*. Mechanically, the nuclear translocation of ALDOA interacts with c-Jun and induces c-Jun phosphorylation at Thr93 by recruiting and enhancing the activation of PAK2, thereby increasing c-Jun transcriptional activity and markedly promoting HCC cell proliferation. Additionally, the AAV-mediated *Aldoa* knockdown specifically suppresses the occurrence and development of HCC in the mouse model of primary HCC. Thus, the regulatory mechanism of ALDOA presented here could have significant implications for the development of novel broad-based anti-cancer therapies.

## CRediT authorship contribution statement

**Xin Yang:** Writing – original draft, Methodology, Investigation, Formal analysis, Data curation. **Guang-Yuan Ma:** Writing – original draft, Methodology, Investigation, Formal analysis, Data curation. **Xiao-Qiang Li:** Writing – original draft, Methodology, Investigation, Formal analysis, Data curation. **Na Tang:** Methodology, Investigation, Formal analysis, Data curation. **Yang Sun:** Methodology, Investigation. **Xiao-Wei Hao:** Methodology, Investigation. **Ke-Han Wu:** Methodology, Investigation. **Yu-Bo Wang:** Methodology, Investigation. **Wen Tian:** Methodology. **Xin Fan:** Methodology. **Zezhi Li:** Methodology. **Caixia Feng:** Methodology. **Xu Chao:** Methodology. **Yu-Fan Wang:** Methodology. **Yao Liu:** Methodology. **Di Li:** Methodology. **Wei Cao:** Writing – review & editing, Supervision, Resources, Project administration, Funding acquisition, Conceptualization.

## Declaration of competing interest

The authors declare that there are no conflicts of interest.
