# Supplementary material for: Aldolase A accelerates hepatocarcinogenesis by refactoring c-Jun transcription
Source: J Pharm Anal. 2024 Dec 16;15(7):101169. doi: 10.1016/j.jpha.2024.101169 (PMC12284681; doi:10.1016/j.jpha.2024.101169)
Supplement: Multimedia component 1 [file mmc1.docx]

**Supplementary Materials**

**Supplementary Figures**


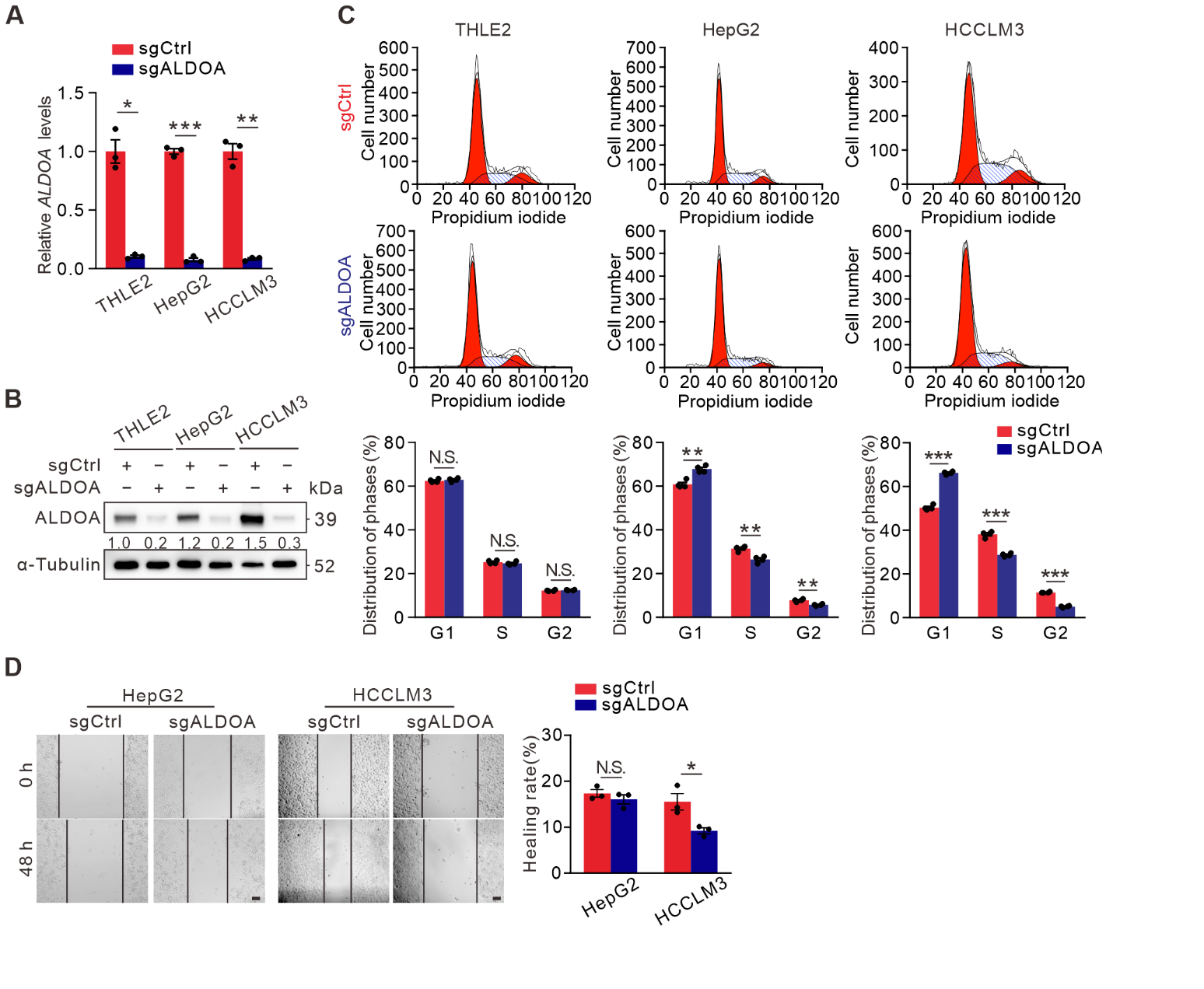


**Fig. S1.** Effect of aldolase A (ALDOA) knockout on tumorigenicity of human liver and hepatocellular carcinoma (HCC) cells *in vitro*. (A-B) *ALDOA* messenger RNA (mRNA) and protein expressions in gene knockout of *ALDOA* (sgALDOA) or nontargeting control (sgCtrl) group of THLE2, HepG2, and HCCLM3 cells (n=3). (C) The effects of *ALDOA* knockout on the cell cycles of THLE2, HepG2, and HCCLM3 cells (n=4). Typical flow cytometric histograms and the percentages (%) of cell subpopulations at different cell cycle stages. (D) Wound healing migration assays showing the influence of *ALDOA* loss on cellular mobility. Scale bars, 100 μm. The representative images were shown in the left panel; The percentages (%) of empty areas were quantified and shown in the right panel (n=3). N.S., no significant difference, ***P*<0.01, ****P*<0.001.


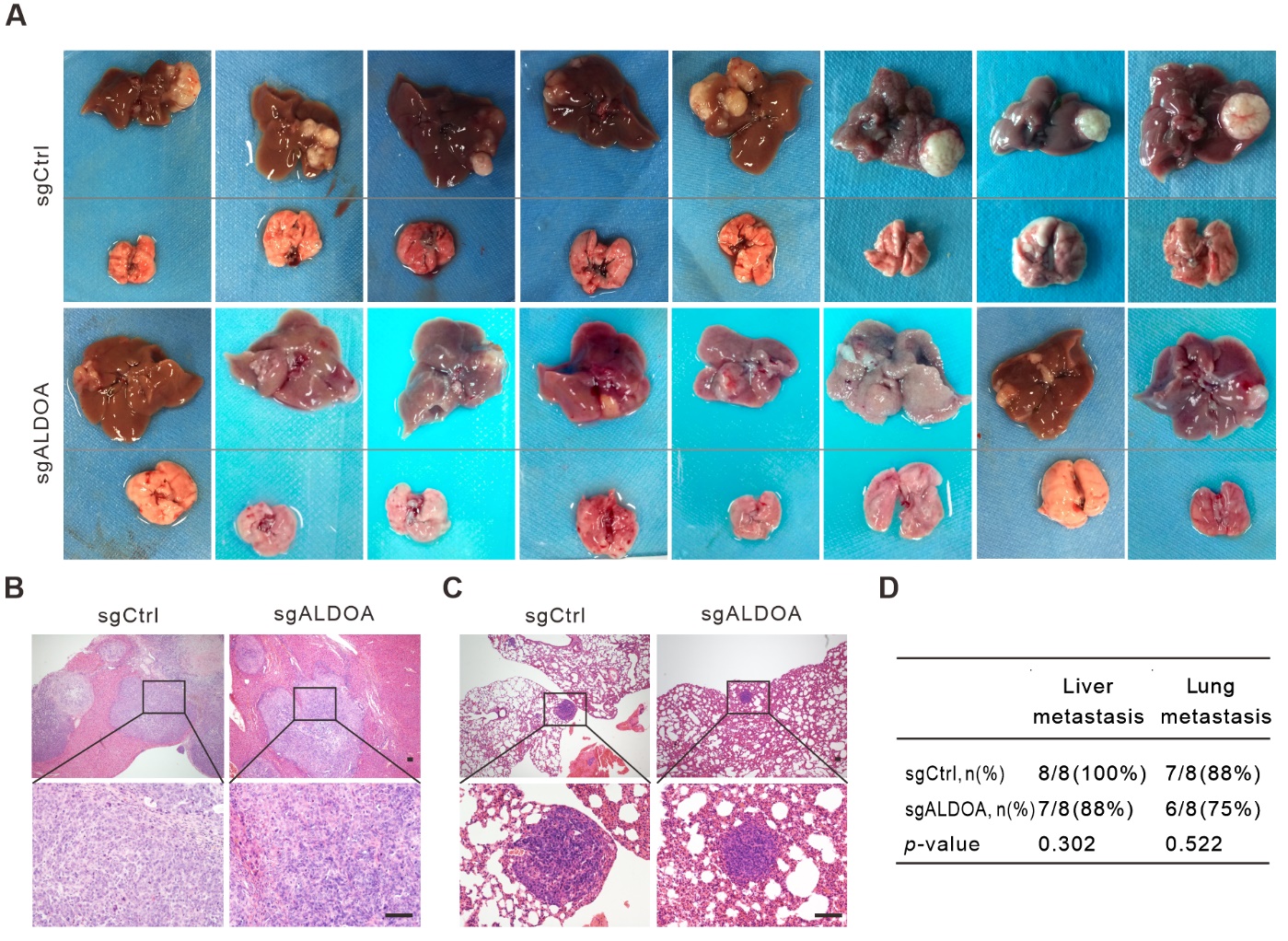


**Fig. S2.** Effect of aldolase A (ALDOA) deficiency on tumorigenicity of HCCLM3 cells in orthotopic xenograft mice. (A) All images of the livers and lungs in orthotopic xenograft mice after transplantation of HCCLM3-gene knockout of *ALDOA* (sgALDOA) or nontargeting control (sgCtrl) cells. (B-D) Representative images of hematoxylin and eosin (H&E) staining for intrahepatic metastatic foci (B) and metastatic lung foci (C), and the numbers of mice with intrahepatic and lung metastases (D) in orthotopic xenograft mice at 42 days (n=8). The boxed area highlights intrahepatic tumor cell proliferation and lung metastasis. Scale bars, 100 μm.


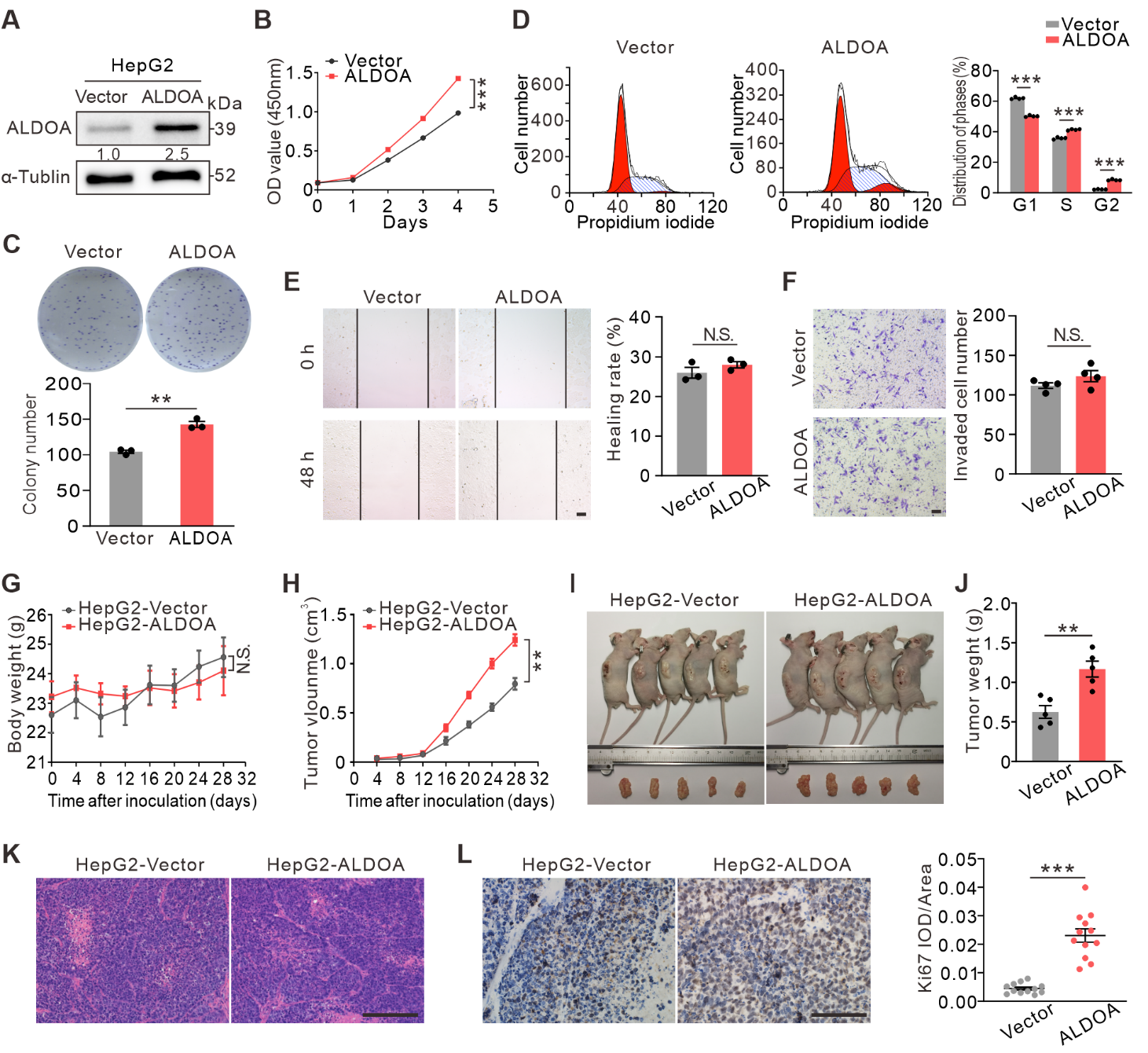


**Fig. S3.** Effect of aldolase A (ALDOA) overexpression on tumorigenicity of HepG2 cells both *in vitro* and *in vivo*. (A) The expression of ALDOA protein in HepG2-*ALDOA* and vector control cells (n=3). (B) Cell proliferation curves of HepG2-*ALDOA* and HepG2-vector cells (n=6). (C) Representative images and quantitative analysis of colony formation of HepG2 cells (n=3). (D) The effects of *ALDOA* overexpression on the cell cycles of HepG2 cells (n=4). Typical flow cytometric histograms (left panel) and cell cycle distribution (right panel). (E) Representative images of wound healing and quantitative analysis (n=4). Scale bars, 100 μm. (F) Representative images and the quantification of transwell matrigel invasion assay (n=4). Scale bars, 100 μm. (G-H) The body weights and tumor volumes of the xenograft mice as monitored every 4 days until 28 days (n=5). (I) The images of xenograft mice and tumor samples at week 4 after transplantation. (J) Tumor weights of the xenograft mice at week 4 after transplantation (n=5). (K) Representative hematoxylin and eosin (H&E) images of the tumor sections. Scale bars, 100 μm. (L) Representative images of Ki67 immunohistochemical staining and quantification of Ki67 expression (n=12). Scale bars, 100 μm. N.S., no significant difference, ***P*<0.01, ****P*<0.001. IOD: integrated optical density.


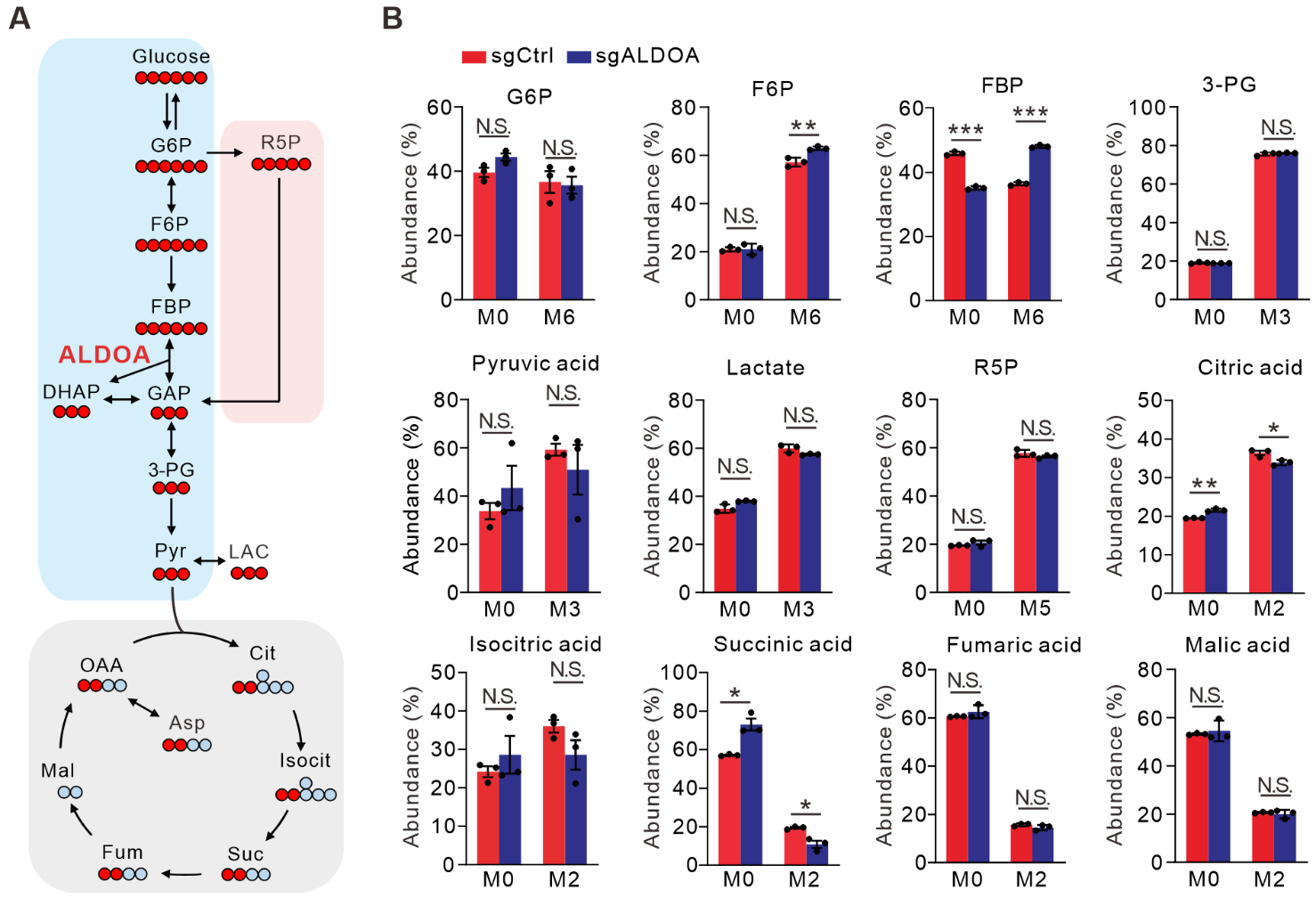


**Fig. S4.** The effect of aldolase A (ALDOA) deficiency on intracellular flux from glucose into lactate and then into the tricarboxylic acid (TCA) cycle. (A) Carbon fate map showing the isotope distribution of indicated metabolites derived from [U-^13^C] glucose. ^13^C atoms are colored in red. (B) Metabolic flux distributions in HCCLM3-gene knockout of *ALDOA* (sgALDOA) and HCCLM3-nontargeting control (sgCtrl) cells. Fluxes were determined by integrating mass isotopic labeling data from [U-^13^C] glucose tracer experiments at 20 h (n=3). N.S., no significant difference, **P*<0.05, ***P*<0.01, ****P*<0.001. G6P: glucose 6-phosphate, R5P: ribulose 5-phosphate, F6P: fructose 6-phosphate, FBP: fructose 1,6-bisphosphate, DHAP: dihydroxyacetone phosphate, GAP: glyceraldehyde 3-phosphate, 3-PG: 3-phosphoglycerate, Pyr: pyruvic acid, LAC: lactate, Cit: citric acid, Isocit: isocitric acid, Suc: succinic acid, Fum: fumaric acid, Mal: malic acid, OAA: oxaloacetic acid, Asp: aspartic acid.


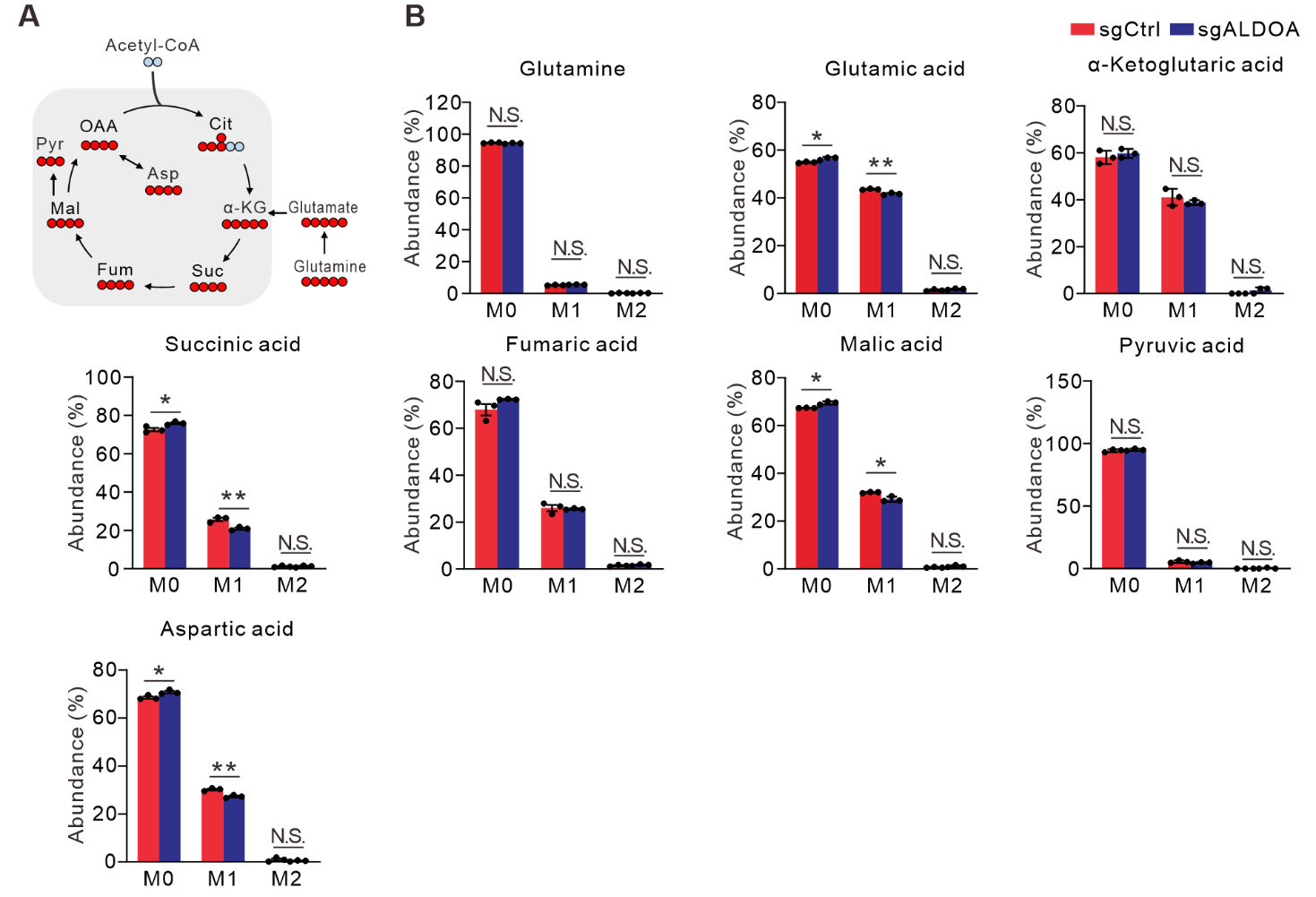


**Fig. S5.** Changes in intracellular glutamine flux by aldolase A (ALDOA) deficiency. (A) Carbon fate map showing the isotope distribution of indicated metabolites derived from [U-^13^C] glutamine. ^13^C atoms are colored in red. (B) Metabolic flux distributions in HCCLM3-gene knockout of *ALDOA* (sgALDOA) and HCCLM3-nontargeting control (sgCtrl) cells. Fluxes were determined by integrating mass isotopic labeling data from [U-^13^C] glutamine tracer experiments at 20 h (n=4). N.S., no significant difference, **P*<0.05, ***P*<0.01. CoA: coenzyme A, Cit: citric acid, α-KG: α-ketoglutaric acid, Suc: succinic acid, Fum: fumaric acid, Mal: malic acid, Pyr: pyruvic acid, OAA: oxaloacetic acid, Asp: aspartic acid.


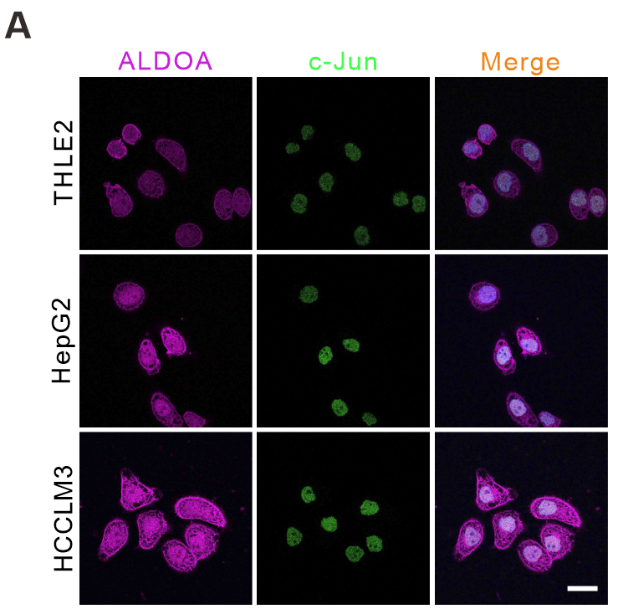


**Fig. S6.** Immunofluorescence microscopic images depicting aldolase A (ALDOA) and c-Jun in three kinds of cells. Scale bars, 10 μm.


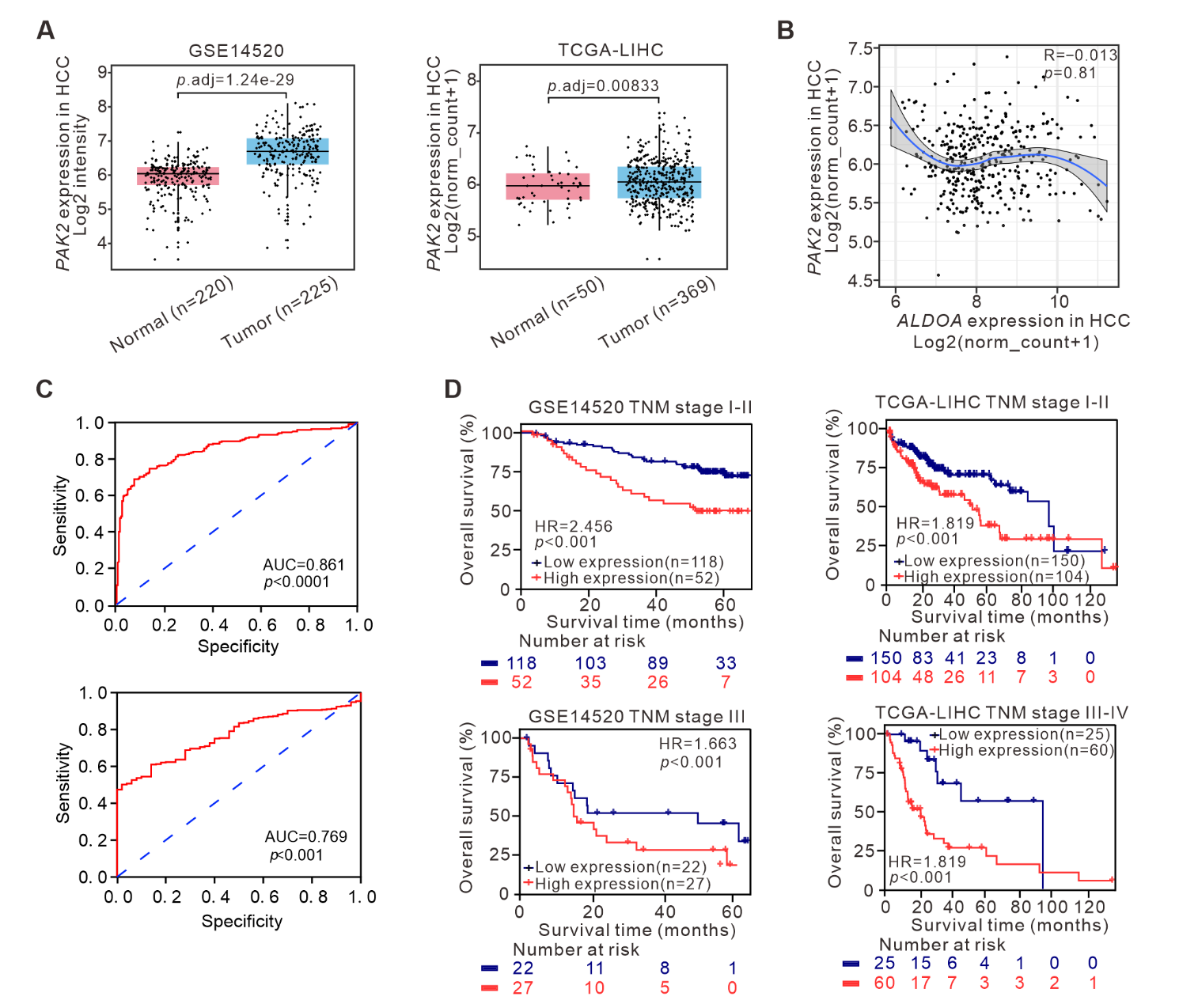


**Fig.** S7. The upregulation of aldolase A (ALDOA) serves as a prognostic marker in hepatocellular carcinoma (HCC) patients. (A) Box plots of *PAK2* messenger RNA (mRNA) expressions in HCC and normal liver tissues from the GSE14520 in Gene Expression Omnibus (GEO) and the Cancer Genome Atlas Liver Hepatocellular Carcinoma (TCGA-LIHC) datasets, respectively. (B) The correlation between *ALDOA* and *PAK2* expressions in TCGA-LIHC datasets. (C) Validation of diagnostic value of *ALDOA* upregulation in HCC using the receiver operating characteristic (ROC) curve in two independent datasets. (D) Survival analysis of *ALDOA* based on tumor-node-metastasis (TNM) I+II or TNM III+IV stages in GSE14520 and TCGA-LIHC datasets. AUC: area under the curve, HR: hazard ratio.


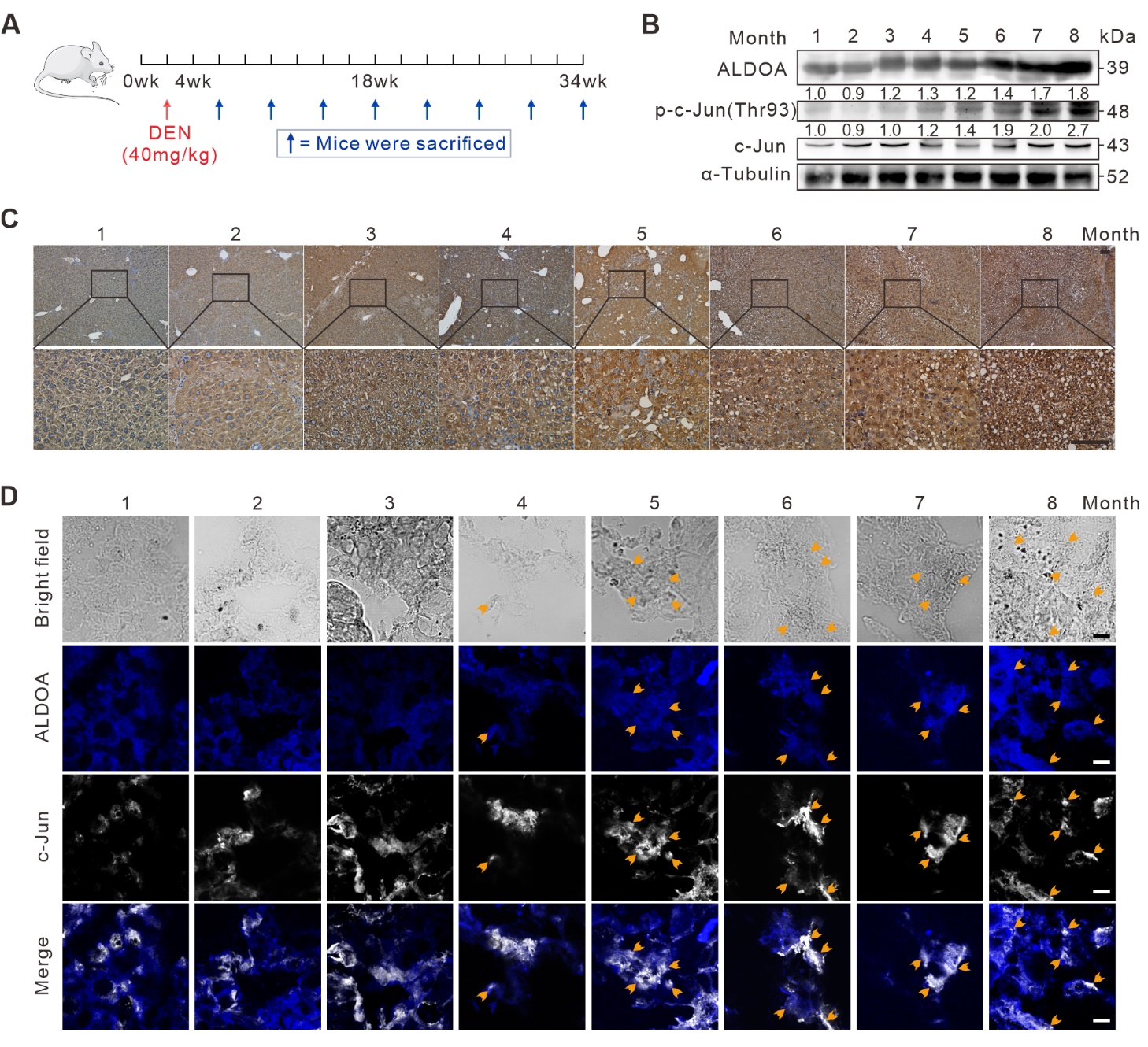


**Fig. S8.** The expressions of aldolase A (ALDOA) and c-Jun in mice livers during diethylnitrosamine (DEN)-induced hepatocellular carcinoma (HCC) formation. (A) Schematic treatment of DEN challenge (intraperitoneally) on male C57BL/6 mice. (B) The protein levels of ALDOA, phosphorylated c-Jun (p-c-Jun) Thr93, and c-Jun in mice livers during HCC modeling (n=3). (C) Representative images of ALDOA immunohistochemistry staining in mice livers of DEN-induced HCC formation. Scale bars, 100 μm. (D) Immunofluorescence analysis of ALDOA and c-Jun in mice livers. Scale bars, 20 μm.


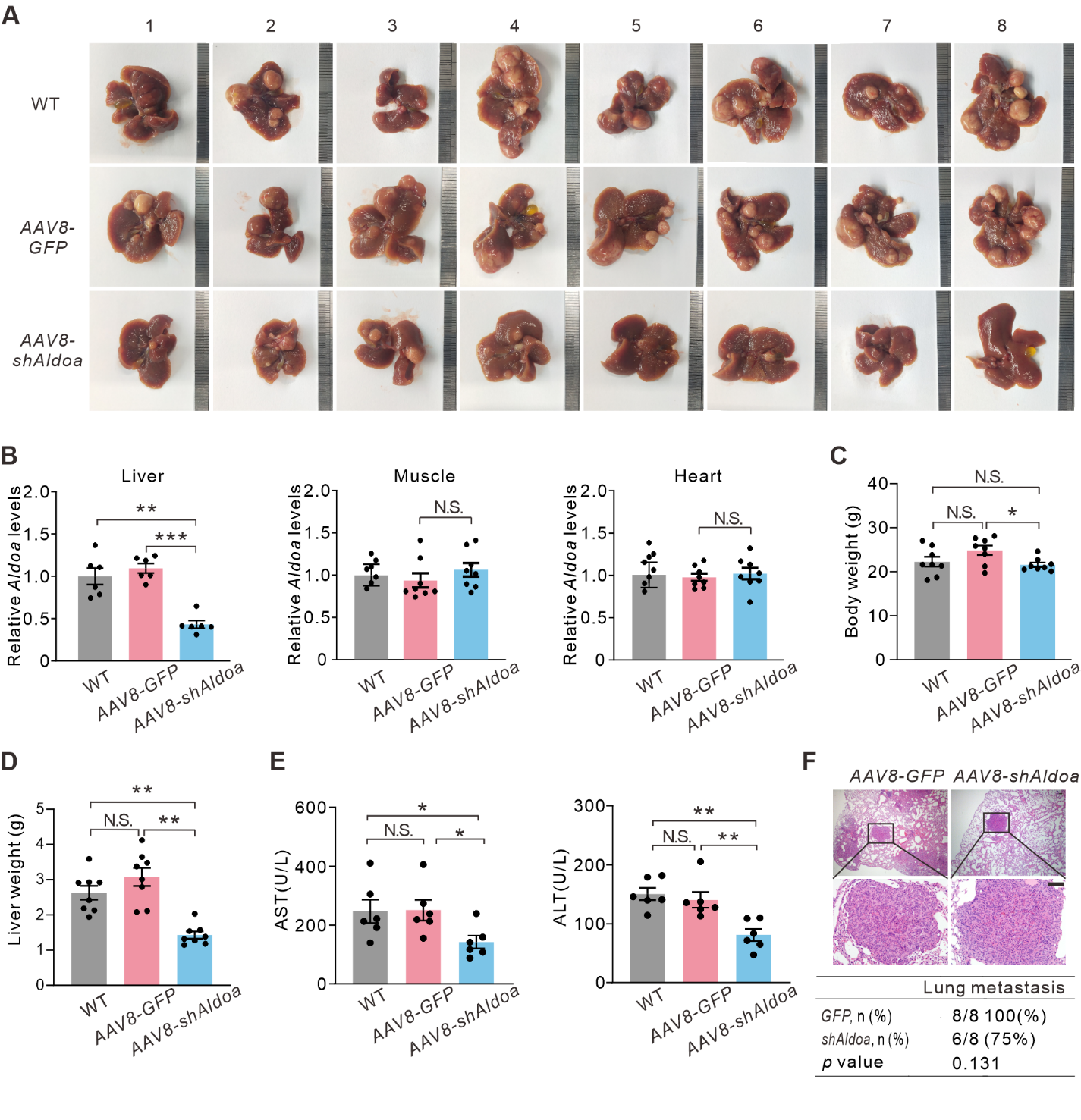


**Fig. S9.** *Aldoa* expression and liver indexes in adeno-associated virus based on serotype 8 (AAV8)-transfected mice after diethylnitrosamine (DEN) treatment. (A) All images of livers at 8 months after DEN treatment. (B) Analysis of *Aldoa* messenger RNA (mRNA) levels in mice's livers, skeletal muscles, and hearts at the time of sacrifice. mRNA data are depicted as relative expression normalized to *Gapdh* (n=6-8). (C-D) The body and liver weights of the animals at the time of sacrifice (n=8). (E) The serum levels of aspartate transaminase (AST) and alanine aminotransferase (ALT) in the DEN-induced mice (n=6). (F) Representative images of hematoxylin and eosin (H&E) staining for metastatic lung foci and the number of lung metastases in each group (n=8). N.S., no significant difference, **P*<0.05, ***P*<0.01, ****P*<0.001. WT: wild-type, *AAV8-GFP*: AAV8 liver-specific GFP control, *AAV8-shAldoa*: AAV8 liver-specific *Aldoa* knockdown.

**Supplementary Tables**

**Table. S1. Primer sets for quantitative real-time PCR (qPCR) analyses**

| **Species** | **Gene symbol** | **Forward (5'-3')** | **Reverse (5'-3')** |
| --- | --- | --- | --- |
| Human | *ALDOA* | ATGCCCTACCAATATCCAGCA | GCTCCCAGTGGACTCATCTG |
|  | *CXCL8* | ACTGAGAGTGATTGAGAGTGGAC | AACCCTCTGCACCCAGTTTTC |
|  | *DUSP1* | ACCTGGGCAGTGCGTATCA | GCCTGGCAGTGGACAAACA |
|  | *FGB* | AGTGATTCAGAACCGTCAAGAC | CATCCTGGTAAGCTGGCTAATTT |
|  | *GAPDH* | GGGAAACTGTGGCGTGAT | GAGTGGGTGTCGCTGTTGA |
|  | *PPP1R15A* | AGCCACGGAGGATAAAAGAACA | CTGAACGATACTCCCAGGACC |
| Murine | *Aldoa* | CGTGTGAATCCCTGCATTGG | CAGCCCCTGGGTAGTTGTC |
|  | *Cxcl1* | CTGGGATTCACCTCAAGAACATC | CAGGGTCAAGGCAAGCCTC |
|  | *Dusp1* | CGCTTCTCGGAAGGATATGCT | GTCAATAGCCTCGTTGAACCAG |
|  | *Fgb* | ACTACGATGAACCGACGGATA | GGCAGGTCTTAGGCTAGGAG |
|  | *Gapdh* | CACTGAGCAAGAGAGGCCCTAT | GCAGCGAACTTTATTGATGGTATT |
|  | *Ppp1r15a* | GAGGGACGCCCACAACTTC | TTACCAGAGACAGGGGTAGGT |

**Table. S2. Complete mass isotope distribution in HCCLM3 cells after knockout of aldolase A (ALDOA)**

| Metabolite name | Retention time  (min) | Mass-to-charge ratio | [U-^13^C]glucose-6 h | | [U-^13^C]glucose-20 h | | [U-^13^C]glutamine-20 h | |
| --- | --- | --- | --- | --- | --- | --- | --- | --- |
|  |  |  | sgCtrl | sgALDOA | sgCtrl | sgALDOA | sgCtrl | sgALDOA |
| Gluc-m0 | 5.8 | 179.0561 | 0.9452 | 0.9428 | 0.9429 | 0.9408 | - | - |
| Gluc-m1 | 5.8 | 180.0595 | 0.0548 | 0.0572 | 0.0571 | 0.0592 | - | - |
| Gluc-m2 | 5.8 | 181.0628 | 0.0000 | 0.0000 | 0.0000 | 0.0000 | - | - |
| Gluc-m3 | 5.8 | 182.0662 | 0.0000 | 0.0000 | 0.0000 | 0.0000 | - | - |
| Gluc-m4 | 5.8 | 183.0695 | 0.0000 | 0.0000 | 0.0000 | 0.0000 | - | - |
| Gluc-m5 | 5.8 | 184.0729 | 0.0000 | 0.0000 | 0.0000 | 0.0000 | - | - |
| Gluc-m6 | 5.8 | 185.0762 | 0.0000 | 0.0000 | 0.0000 | 0.0000 | - | - |
| G6P-m0 | 6.1 | 259.0224 | 0.3578 | 0.5162 | 0.3962 | 0.4445 | - | - |
| G6P-m1 | 6.1 | 260.0258 | 0.0063 | 0.0044 | 0.0136 | 0.0109 | - | - |
| G6P-m2 | 6.1 | 261.0292 | 0.0000 | 0.0000 | 0.0000 | 0.0000 | - | - |
| G6P-m3 | 6.1 | 262.0325 | 0.0050 | 0.0045 | 0.0158 | 0.0101 | - | - |
| G6P-m4 | 6.1 | 263.0359 | 0.0000 | 0.0000 | 0.0000 | 0.0000 | - | - |
| G6P-m5 | 6.1 | 264.0392 | 0.2585 | 0.1763 | 0.2075 | 0.1777 | - | - |
| G6P-m6 | 6.1 | 265.0426 | 0.3724 | 0.2987 | 0.3669 | 0.3569 | - | - |
| F6P-m0 | 5.89 | 259.0224 | 0.3827 | 0.2851 | 0.2098 | 0.2105 | - | - |
| F6P-m1 | 5.89 | 260.0258 | 0.0227 | 0.0173 | 0.0099 | 0.0117 | - | - |
| F6P-m2 | 5.89 | 261.0292 | 0.0127 | 0.0039 | 0.0078 | 0.0064 | - | - |
| F6P-m3 | 5.89 | 262.0325 | 0.0431 | 0.0392 | 0.1055 | 0.0704 | - | - |
| F6P-m4 | 5.89 | 263.0359 | 0.0324 | 0.0199 | 0.0280 | 0.0152 | - | - |
| F6P-m5 | 5.89 | 264.0392 | 0.0525 | 0.0519 | 0.0673 | 0.0562 | - | - |
| F6P-m6 | 5.89 | 265.0426 | 0.4538 | 0.5827 | 0.5717 | 0.6297 | - | - |
| FBP-m0 | 6.4 | 338.9888 | 0.6635 | 0.2878 | 0.4594 | 0.3514 | - | - |
| FBP-m1 | 6.4 | 339.9921 | 0.0372 | 0.0192 | 0.0288 | 0.0222 | - | - |
| FBP-m2 | 6.4 | 340.9955 | 0.0030 | 0.0092 | 0.0035 | 0.0065 | - | - |
| FBP-m3 | 6.4 | 341.9988 | 0.0319 | 0.0521 | 0.0812 | 0.0767 | - | - |
| FBP-m4 | 6.4 | 343.0022 | 0.0057 | 0.0170 | 0.0158 | 0.0164 | - | - |
| FBP-m5 | 6.4 | 344.0055 | 0.0228 | 0.0530 | 0.0467 | 0.0456 | - | - |
| FBP-m6 | 6.4 | 345.0089 | 0.2359 | 0.5617 | 0.3647 | 0.4812 | - | - |
| R5P-m0 | 5.72 | 229.0119 | 0.4780 | 0.4848 | 0.1949 | 0.2060 | - | - |
| R5P-m1 | 5.72 | 230.0152 | 0.0244 | 0.0312 | 0.0184 | 0.0140 | - | - |
| R5P-m2 | 5.72 | 231.0186 | 0.0494 | 0.0621 | 0.1419 | 0.1194 | - | - |
| R5P-m3 | 5.72 | 232.0219 | 0.0182 | 0.0179 | 0.0363 | 0.0284 | - | - |
| R5P-m4 | 5.72 | 233.0253 | 0.0407 | 0.0451 | 0.0814 | 0.0689 | - | - |
| R5P-m5 | 5.72 | 234.0287 | 0.3893 | 0.3588 | 0.5771 | 0.5633 | - | - |
| 3PG-m0 | 5.92 | 184.9857 | 0.1720 | 0.1803 | 0.1909 | 0.1885 | - | - |
| 3PG-m1 | 5.92 | 185.989 | 0.0106 | 0.0139 | 0.0178 | 0.0150 | - | - |
| 3PG-m2 | 5.92 | 186.9924 | 0.0427 | 0.0399 | 0.0413 | 0.0360 | - | - |
| 3PG-m3 | 5.92 | 187.9957 | 0.7747 | 0.7659 | 0.7500 | 0.7605 | - | - |
| Lact-m0 | 1.65 | 89.0244 | 0.5618 | 0.5223 | 0.3482 | 0.3794 | - | - |
| Lact-m1 | 1.65 | 90.0278 | 0.0251 | 0.0235 | 0.0194 | 0.0182 | - | - |
| Lact-m2 | 1.65 | 91.0311 | 0.0228 | 0.0241 | 0.0334 | 0.0282 | - | - |
| Lact-m3 | 1.65 | 92.0345 | 0.3902 | 0.4301 | 0.5990 | 0.5742 | - | - |
| Pyr-m0 | 174 | 6.25 | - | - | 0.3374 | 0.4338 | 0.9435 | 0.9510 |
| Pyr-m1 | 175 | 6.25 | - | - | 0.0377 | 0.0258 | 0.0560 | 0.0464 |
| Pyr-m2 | 176 | 6.25 | - | - | 0.0326 | 0.0314 | 0.0006 | 0.0026 |
| Pyr-m3 | 177 | 6.25 | - | - | 0.5922 | 0.5090 | 0.0000 | 0.0000 |
| Cit-m0 | 591 | 16.9 | - | - | 0.1948 | 0.2157 | 0.6544 | 0.6746 |
| Cit-m1 | 592 | 16.9 | - | - | 0.0526 | 0.0687 | 0.3244 | 0.3047 |
| Cit-m2 | 593 | 16.9 | - | - | 0.3641 | 0.3387 | 0.0177 | 0.0190 |
| Cit-m3 | 594 | 16.9 | - | - | 0.1128 | 0.1158 | 0.0016 | 0.0015 |
| Cit-m4 | 595 | 16.9 | - | - | 0.1446 | 0.1533 | 0.0018 | 0.0000 |
| Cit-m5 | 596 | 16.9 | - | - | 0.0808 | 0.0815 | 0.0000 | 0.0000 |
| Cit-m6 | 597 | 16.9 | - | - | 0.0498 | 0.0256 | 0.0000 | 0.0001 |
| Isocit-m0 | 591 | 16.94 | - | - | 0.2417 | 0.2858 | 0.6509 | 0.6384 |
| Isocit-m1 | 592 | 16.94 | - | - | 0.0673 | 0.0251 | 0.3045 | 0.3336 |
| Isocit-m2 | 593 | 16.94 | - | - | 0.3605 | 0.2858 | 0.0433 | 0.0170 |
| Isocit-m3 | 594 | 16.94 | - | - | 0.0783 | 0.1875 | 0.0013 | 0.0103 |
| Isocit-m4 | 595 | 16.94 | - | - | 0.1548 | 0.1205 | 0.0000 | 0.0007 |
| Isocit-m5 | 596 | 16.94 | - | - | 0.0950 | 0.0736 | 0.0000 | 0.0000 |
| Isocit-m6 | 597 | 16.94 | - | - | 0.0024 | 0.0218 | 0.0000 | 0.0000 |
| Suc-m0 | 289 | 11.95 | - | - | 0.5711 | 0.7309 | 0.7255 | 0.7584 |
| Suc-m1 | 290 | 11.95 | - | - | 0.1023 | 0.0856 | 0.2575 | 0.2093 |
| Suc-m2 | 291 | 11.95 | - | - | 0.1942 | 0.1082 | 0.0122 | 0.0124 |
| Suc-m3 | 292 | 11.95 | - | - | 0.0844 | 0.0604 | 0.0025 | 0.0024 |
| Suc-m4 | 293 | 11.95 | - | - | 0.0381 | 0.0250 | 0.0024 | 0.0041 |
| Fum-m0 | 287 | 12.23 | - | - | 0.6062 | 0.6260 | 0.6794 | 0.7229 |
| Fum-m1 | 288 | 12.23 | - | - | 0.1044 | 0.1089 | 0.2599 | 0.2561 |
| Fum-m2 | 289 | 12.23 | - | - | 0.1578 | 0.1460 | 0.0139 | 0.0170 |
| Fum-m3 | 290 | 12.23 | - | - | 0.0991 | 0.0899 | 0.0038 | 0.0030 |
| Fum-m4 | 291 | 12.23 | - | - | 0.0325 | 0.0292 | 0.0430 | 0.0009 |
| Mal-m0 | 419 | 14.45 | - | - | 0.5321 | 0.5455 | 0.6735 | 0.6945 |
| Mal-m1 | 420 | 14.45 | - | - | 0.1027 | 0.1021 | 0.3196 | 0.2916 |
| Mal-m2 | 421 | 14.45 | - | - | 0.2064 | 0.1996 | 0.0063 | 0.0108 |
| Mal-m3 | 422 | 14.45 | - | - | 0.1157 | 0.1154 | 0.0005 | 0.0025 |
| Mal-m4 | 423 | 14.45 | - | - | 0.0432 | 0.0374 | 0.0002 | 0.0005 |
| Asp-m0 | 418 | 14.73 | - | - | 0.5710 | 0.5648 | 0.6858 | 0.7086 |
| Asp-m1 | 419 | 14.73 | - | - | 0.1088 | 0.0933 | 0.3022 | 0.2728 |
| Asp-m2 | 420 | 14.73 | - | - | 0.1758 | 0.1939 | 0.0092 | 0.0044 |
| Asp-m3 | 421 | 14.73 | - | - | 0.1143 | 0.1144 | 0.0000 | 0.0042 |
| Asp-m4 | 422 | 14.73 | - | - | 0.0301 | 0.0336 | 0.0029 | 0.0000 |
| Glu-m0 | 432 | 15.44 | - | - | 0.5225 | 0.5207 | 0.5487 | 0.5649 |
| Glu-m1 | 433 | 15.44 | - | - | 0.0794 | 0.0801 | 0.4362 | 0.4165 |
| Glu-m2 | 434 | 15.44 | - | - | 0.2173 | 0.2139 | 0.0132 | 0.0177 |
| Glu-m3 | 435 | 15.44 | - | - | 0.0843 | 0.0858 | 0.0014 | 0.0004 |
| Glu-m4 | 436 | 15.44 | - | - | 0.0695 | 0.0715 | 0.0006 | 0.0000 |
| Glu-m5 | 437 | 15.44 | - | - | 0.0270 | 0.0280 | 0.0000 | 0.0004 |
| Gln-m0 | 431 | 16.19 | - | - | 0.9271 | 0.9264 | 0.0000 | 0.0000 |
| Gln-m1 | 432 | 16.19 | - | - | 0.0628 | 0.0668 | 0.9460 | 0.9422 |
| Gln-m2 | 433 | 16.19 | - | - | 0.0069 | 0.0043 | 0.0519 | 0.0552 |
| Gln-m3 | 434 | 16.19 | - | - | 0.0019 | 0.0017 | 0.0019 | 0.0022 |
| Gln-m4 | 435 | 16.19 | - | - | 0.0013 | 0.0004 | 0.0002 | 0.0000 |
| Gln-m5 | 436 | 16.19 | - | - | 0.0000 | 0.0004 | 0.0001 | 0.0004 |
| α-KG-m0 | 346 | 13.79 | - | - | 0.5345 | 0.4804 | 0.5810 | 0.5979 |
| α-KG-m1 | 347 | 13.79 | - | - | 0.0773 | 0.0430 | 0.4108 | 0.3879 |
| α-KG-m2 | 348 | 13.79 | - | - | 0.1814 | 0.2610 | 0.0001 | 0.0143 |
| α-KG-m3 | 349 | 13.79 | - | - | 0.0311 | 0.0995 | 0.0081 | 0.0000 |
| α-KG-m4 | 350 | 13.79 | - | - | 0.0895 | 0.0568 | 0.0000 | 0.0000 |
| α-KG-m5 | 351 | 13.79 | - | - | 0.0862 | 0.0593 | 0.0000 | 0.0000 |
| Ser-m0 | 13.64 | 390 | - | - | 0.8624 | 0.9027 | 0.9948 | 0.9903 |
| Ser-m1 | 13.64 | 391 | - | - | 0.0463 | 0.0381 | 0.0039 | 0.0046 |
| Ser-m2 | 13.64 | 392 | - | - | 0.0343 | 0.0190 | 0.0009 | 0.0018 |
| Ser-m3 | 13.64 | 393 | - | - | 0.0570 | 0.0401 | 0.0004 | 0.0033 |
| Gly-m0 | 10.22 | 246 | - | - | 0.9321 | 0.9444 | 0.9756 | 0.9703 |
| Gly-m1 | 10.22 | 247 | - | - | 0.0296 | 0.0283 | 0.0223 | 0.0272 |
| Gly-m2 | 10.22 | 248 | - | - | 0.0383 | 0.0273 | 0.0021 | 0.0025 |

**Notes:** sgCtrl: nontargeting control, sgALDOA: gene knockout of *ALDOA*, Gluc: glucose, G6P: glucose 6-phosphate, F6P: fructose 6-phosphate, FBP: fructose 1,6-bisphosphate, R5P: ribulose 5-phosphate, 3-PG: 3-phosphoglycerate, Lact: lactate, Pyr: pyruvic acid, Cit: citric acid, Isocit: isocitric acid, Suc: succinic acid, Fum: fumaric acid, Mal: malic acid, Asp: aspartic acid, Glu: glutamic acid, Gln: glutamine, α-KG: α-ketoglutaric acid, Ser: serine, Gly: glycine.

**Table. S3. Top up-regulated and down-regulated genes in HCCLM3 cells after knockout of aldolase A (ALDOA)**

| Gene Symbol | Description | Fold change  (vs control, log2) | *p*-value |
| --- | --- | --- | --- |
| *MPZL1* | Myelin protein zero-like protein 1 | 1.4537 | 3.2375E-06 |
| *BBX* | HMG box transcription factor BBX | 1.1266 | 0.0081 |
| *INSIG1* | Insulin-induced gene 1 protein | 1.0596 | 0.0060 |
| *RP11-407N17.3* | cTAGE family member 5 | 1.0048 | 0.0312 |
| *NR0B2* | Nuclear receptor subfamily 0 group B member 2 | 0.9483 | 0.0474 |
| *CLDND2* | Claudin domain containing 2 | 0.9189 | 0.0028 |
| *GATA4* | Transcription factor GATA-4 | 0.8274 | 0.0191 |
| *CLCN5* | Chloride voltage-gated channel 5 | 0.8203 | 0.0216 |
| *VAMP3* | Vesicle-associated membrane protein 3 | 0.8168 | 0.0000 |
| *EPHB2* | Ephrin type-B receptor 2 | 0.7804 | 0.0041 |
| *ZNF155* | Zinc finger protein 155 | 0.7604 | 0.0353 |
| *KDR* | Kinase insert domain receptor | 0.7594 | 0.0167 |
| *SHTN1* | Shootin-1 | 0.7579 | 0.0001 |
| *TGFBR1* | Transforming growth factor beta receptor 1 | 0.7462 | 0.0469 |
| *FADS2* | Fatty acid desaturase 2 | 0.7379 | 0.0063 |
| *PPM1M* | Protein phosphatase, Mg^2+^/Mn^2+^ dependent 1M | 0.7176 | 0.0025 |
| *HIST2H4B* | Histone cluster 2 H4 family member b | 0.7128 | 0.0111 |
| *SPANXA1* | Sperm protein associated with the nucleus | 0.7111 | 0.0115 |
| *LAD1* | Ladinin-1 | 0.7038 | 0.0006 |
| *SLC28A2* | Sodium/nucleoside cotransporter 2 | 0.7023 | 0.0029 |
| *MLLT11* | Myeloid/lymphoid or mixed-lineage leukemia; translocated to, 11 | 0.6980 | 0.0017 |
| *PSKH1* | Protein serine kinase H1 | 0.6846 | 0.0043 |
| *SCD* | Acyl-CoA desaturase | 0.6731 | 0.0142 |
| *SPANXA2* | SPANX family member A2 | 0.6720 | 0.0072 |
| *SPANXC* | Sperm protein associated with the nucleus on the X chromosome C | 0.6719 | 0.0022 |
| *KRT19* | Keratin, type I cytoskeletal 19 | 0.6641 | 0.0005 |
| *TSPAN8* | Tetraspanin-8 | 0.6544 | 0.0250 |
| *ANXA10* | Annexin A10 | 0.6501 | 0.0018 |
| *RNASE1* | Ribonuclease A family member 1, pancreatic | 0.6458 | 0.0174 |
| *COL5A3* | Collagen alpha-3(V) chain | 0.6341 | 0.0230 |
| *GCKR* | Glucokinase regulatory protein | 0.6340 | 0.0087 |
| *FNDC4* | Fibronectin type III domain-containing protein 4 | 0.6204 | 0.0403 |
| *TMEM135* | Transmembrane protein 135 | 0.6171 | 0.0069 |
| *PCSK9* | Proprotein convertase subtilisin/kexin type 9 | 0.6052 | 0.0194 |
| *CALB2* | Calbindin 2 | 0.6036 | 0.0201 |
| *RP11-134F2.8* | - | 0.5989 | 0.0483 |
| *ALDOA* | Fructose-bisphosphate aldolase A | -1.9235 | 4.7318E-06 |
| *EGR1* | Early growth response protein 1 | -1.8004 | 0.0059 |
| *RNF185* | E3 ubiquitin-protein ligase RNF185 | -1.2122 | 0.0361 |
| *CXCL8* | Interleukin-8 | -1.0737 | 0.0320 |
| *DUSP1* | Dual specificity protein phosphatase 1 | -1.0421 | 0.0275 |
| *FOS* | Proto-oncogene c-Fos | -0.9750 | 0.0063 |
| *PCDHB2* | Protocadherin beta-2 | -0.9414 | 0.0021 |
| *AP000295.9* | - | -0.8754 | 0.0362 |
| *FGB* | Fibrinogen beta chain | -0.8347 | 0.0224 |
| *FBXO31* | F-box only protein 31 | -0.8255 | 8.1915E-05 |
| *PPP1R15A* | Protein phosphatase 1 regulatory subunit 15A | -0.8219 | 0.0257 |
| *CTGF* | Connective tissue growth factor | -0.8098 | 0.0214 |
| *TM4SF19-TCTEX1D2* | TM4SF19-DYNLT2B readthrough (NMD Candidate) | -0.7905 | 0.0066 |
| *EID3* | EP300-interacting inhibitor of differentiation 3 | -0.7591 | 0.0383 |
| *SCAMP1* | Secretory carrier-associated membrane protein 1 | -0.7374 | 0.0413 |
| *CISD2* | CDGSH iron-sulfur domain-containing protein 2 | -0.7163 | 0.0038 |
| *UGT2A3* | UDP-glucuronosyltransferase 2A3 | -0.6949 | 0.0142 |
| *IER3* | Immediate early response 3 | -0.6922 | 0.0108 |
| *DPH2* | Diphthamide biosynthesis 2 | -0.6866 | 0.0189 |
| *ATP5J2-PTCD1* | ATP5MF-PTCD1 readthrough | -0.6701 | 0.0160 |
| *PEX2* | Peroxisome biogenesis factor 2 | -0.6603 | 0.0156 |
| *SERTAD3* | SERTA domain-containing protein 3 | -0.6555 | 0.0437 |
| *DUSP6* | Dual specificity protein phosphatase 6 | -0.6527 | 0.0047 |
| *AMOTL2* | Angiomotin-like protein 2 | -0.6275 | 0.0054 |
| *WDR89* | WD repeat-containing protein 89 | -0.6258 | 0.0008 |
| *TMPRSS2* | Transmembrane protease serine 2 | -0.6185 | 0.0007 |
| *ARCN1* | Archain 1 | -0.6131 | 0.0093 |
| *MTRNR2L8* | MT-RNR2 like 8 | -0.6027 | 0.0295 |
| *SGSH* | N-sulphoglucosamine sulphohydrolase | -0.6021 | 0.0471 |
| *ITM2A* | Integral membrane protein 2A | -0.5958 | 0.0003 |

**Table. S4. Association between aldolase A (ALDOA) expression and the clinical parameters in hepatocellular carcinoma (HCC) patients in GSE14520 datasets**

| Parameter | Total (N=221) | *ALDOA* expression | | *p*-value |
| --- | --- | --- | --- | --- |
|  |  | **High**  **(N=70)** | **Low**  **(N=151)** |  |
| Age (year) |  |  |  |  |
| < 65 | 196 (88.7%) | 65 (92.9%) | 131 (86.8%) | 0.27 |
| ≥ 65 | 25 (11.3%) | 5 (7.1%) | 20 (13.2%) |  |
| Gender |  |  |  |  |
| Male | 191 (86.4%) | 65 (92.9%) | 126 (83.4%) | 0.0911 |
| Female | 30 (13.6%) | 5 (7.1%) | 25 (16.6%) |  |
| AFP (ng/mL) |  |  |  |  |
| <300 | 118 (53.4%) | 31 (44.3%) | 87 (57.6%) | 0.179 |
| ≥300 | 100 (45.2%) | 38 (54.3%) | 62 (41.1%) |  |
| Unknown | 3 (1.4%) | 1 (1.4%) | 2 (1.3%) |  |
| ALT (50U/L) |  |  |  |  |
| <50 | 130 (58.8%) | 33 (47.1%) | 97 (64.2%) | 0.0241 |
| ≥50 | 91 (41.2%) | 37 (52.9%) | 54 (35.8%) |  |
| Cirrhosis |  |  |  |  |
| Yes | 203 (91.9%) | 68 (97.1%) | 135 (89.4%) | 0.0906 |
| No | 18 (8.1%) | 2 (2.9%) | 16 (10.6%) |  |
| Main tumor size (cm) |  |  |  |  |
| <5 | 140 (63.3%) | 34 (48.6%) | 106 (70.2%) | 0.00509 |
| ≥5 | 80 (36.2%) | 36 (51.4%) | 44 (29.1%) |  |
| Unknown | 1 (0.5%) | 0 (0%) | 1 (0.7%) |  |
| Multinodular |  |  |  |  |
| Yes | 45 (20.4%) | 19 (27.1%) | 26 (17.2%) | 0.127 |
| No | 176 (79.6%) | 51 (72.9%) | 125 (82.8%) |  |
| TNM stage |  |  |  |  |
| I | 93 (42.1%) | 18 (25.7%) | 75 (49.7%) | <0.001 |
| II | 77 (34.8%) | 24 (34.3%) | 53 (35.1%) |  |
| III | 49 (22.2%) | 27 (38.6%) | 22 (14.6%) |  |
| Unknown | 2 (0.9%) | 1 (1.4%) | 1 (0.7%) |  |
| BCLC stage |  |  |  |  |
| 0 | 20 (9.0%) | 6 (8.6%) | 14 (9.3%) | 0.00537 |
| A | 148 (67.0%) | 38 (54.3%) | 110 (72.8%) |  |
| B/C | 22 (10.0%) | 8 (11.4%) | 14 (9.3%) |  |
| Unknown | 31 (14.0%) | 18 (25.7%) | 13 (8.6%) |  |
| CLIP stage |  |  |  |  |
| 0 | 97 (43.9%) | 23 (32.9%) | 74 (49.0%) | 0.0291 |
| 1 | 74 (33.5%) | 23 (32.9%) | 51 (33.8%) |  |
| 2-5 | 48 (21.7%) | 23 (32.9%) | 25 (16.6%) |  |
| Unknown | 2 (0.9%) | 1 (1.4%) | 1 (0.7%) |  |
| Living status |  |  |  |  |
| Alive | 136 (61.5%) | 29 (41.4%) | 107 (70.9%) | <0.001 |
| Dead | 85 (38.5%) | 41 (58.6%) | 44 (29.1%) |  |

**Notes:** AFP: alpha-fetoprotein, ALT: alanine aminotransferase, TNM: tumor-node-metastasis, BCLC: Barcelona Clinic Liver Cancer, CLIP: Cancer of the Liver Italian Program.

**Table. S5. Association between aldolase A (ALDOA) expression and the clinical parameters in HCC patients in the Cancer Genome Atlas Liver Hepatocellular Carcinoma (TCGA-LIHC) datasets**

| Parameter | Total  (N=363) | *ALDOA* expression | | *p*-value |
| --- | --- | --- | --- | --- |
|  |  | **High**  **(N=214)** | **Low**  **(N=149)** |  |
| Age (year) |  |  |  |  |
| < 65 | 216 (59.5%) | 127 (59.3%) | 89 (59.7%) | 1 |
| ≥ 65 | 147 (40.5%) | 87 (40.7%) | 60 (40.3%) |  |
| Gender |  |  |  |  |
| Male | 245 (67.5%) | 141 (65.9%) | 104 (69.8%) | 0.504 |
| Female | 118 (32.5%) | 73 (34.1%) | 45 (30.2%) |  |
| Family history of cancer |  |  |  |  |
| No | 204 (56.2%) | 123 (57.5%) | 81 (54.4%) | 0.666 |
| Yes | 110 (30.3%) | 61 (28.5%) | 49 (32.9%) |  |
| Unknown | 49 (13.5%) | 30 (14.0%) | 19 (12.8%) |  |
| TNM stage |  |  |  |  |
| I | 170 (46.8%) | 84 (39.3%) | 86 (57.7%) | 0.00713 |
| II | 84 (23.1%) | 56 (26.2%) | 28 (18.8%) |  |
| III | 81 (22.3%) | 55 (25.7%) | 26 (17.4%) |  |
| IV | 4 (1.1%) | 4 (1.9%) | 0 (0%) |  |
| Unknown | 24 (6.6%) | 15 (7.0%) | 9 (6.0%) |  |
| Histologic grade |  |  |  |  |
| G1-2 | 230 (63.4%) | 131 (61.2%) | 99 (66.4%) | 0.348 |
| G3-4 | 128 (35.3%) | 81 (37.9%) | 47 (31.5%) |  |
| Unknown | 5 (1.4%) | 2 (0.9%) | 3 (2.0%) |  |
| Ishak score |  |  |  |  |
| 0-4 | 132 (36.4%) | 67 (31.3%) | 65 (43.6%) | 0.0132 |
| 5-6 | 77 (21.2%) | 43 (20.1%) | 34 (22.8%) |  |
| Unknown | 154 (42.4%) | 104 (48.6%) | 50 (33.6%) |  |
| Child-pugh grade |  |  |  |  |
| A | 216 (59.5%) | 117 (54.7%) | 99 (66.4%) | 0.0204 |
| B-C | 22 (6.1%) | 11 (5.1%) | 11 (7.4%) |  |
| Unknown | 125 (34.4%) | 86 (40.2%) | 39 (26.2%) |  |
| Vascular invasion |  |  |  |  |
| None | 205 (56.5%) | 108 (50.5%) | 97 (65.1%) | 0.0515 |
| Micro | 90 (24.8%) | 60 (28.0%) | 30 (20.1%) |  |
| Macro | 14 (3.9%) | 9 (4.2%) | 5 (3.4%) |  |
| Unknown | 54 (14.9%) | 37 (17.3%) | 17 (11.4%) |  |
| Alpha fetoprotein |  |  |  |  |
| Negative | 147 (40.5%) | 79 (36.9%) | 68 (45.6%) | 0.246 |
| Positive | 129 (35.5%) | 80 (37.4%) | 49 (32.9%) |  |
| Unknown | 87 (24.0%) | 55 (25.7%) | 32 (21.5%) |  |
| Residual tumor |  |  |  |  |
| R0 | 320 (88.2%) | 188 (87.9%) | 132 (88.6%) | 0.89 |
| R1-R2 | 16 (4.4%) | 9 (4.2%) | 7 (4.7%) |  |
| Unknown | 27 (7.4%) | 17 (7.9%) | 10 (6.7%) |  |
| Living status |  |  |  |  |
| Alive | 234 (64.5%) | 125 (58.4%) | 109 (73.2%) | 0.00551 |
| Dead | 129 (35.5%) | 89 (41.6%) | 40 (26.8%) |  |
| Disease status |  |  |  |  |
| No | 170 (46.8%) | 96 (44.9%) | 74 (49.7%) | 0.594 |
| Yes | 143 (39.4%) | 86 (40.2%) | 57 (38.3%) |  |
| Unknown | 50 (13.8%) | 32 (15.0%) | 18 (12.1%) |  |

**Notes:** TNM: tumor-node-metastasis.

**Table. S6. Univariate and multivariate Cox regression analysis of overall survival in GSE14520 datasets**

| Variable | Univariate analysis | | Multivariate analysis | |
| --- | --- | --- | --- | --- |
|  | **Hazard ratio (95% CI)** | ***p*-value** | **Hazard ratio (95% CI)** | ***p*-value** |
| Age (≥ 65 vs. < 65) | 0.54 (0.24,1.24) | 0.148 | - | - |
| Gender (Female vs. Male) | 1.7 (0.82,3.52) | 0.153 | - | - |
| AFP (≥ 300 vs. < 300 ng/mL) | 1.63 (1.06,2.5) | 0.025 | 1.15 (0.58,2.26) | 0.694 |
| ALT (≥ 50 vs. < 50 U/L) | 1.08 (0.7,1.66) | 0.727 | - | - |
| Cirrhosis (Yes vs. No) | 4.62 (1.14,18.8) | 0.032 | 4.16 (1,17.36) | 0.050 |
| Main tumor size (≥ 5 vs. < 5 cm) | 1.92 (1.25,2.96) | 0.003 | 0.93 (0.54,1.61) | 0.806 |
| Multinodular (Yes vs. No) | 1.59 (0.99,2.57) | 0.057 | - | - |
| TNM stage (II vs. I) | 2.14 (1.24,3.72) | 0.007 | 1.8 (1.01,3.19) | 0.045 |
| TNM stage (III vs. I) | 5.21 (2.97,9.14) | <0.001 | 2.01 (0.84,4.78) | 0.116 |
| BCLC stage (A vs. 0) | 4.09 (0.99,16.82) | 0.051 | 3.98 (0.95,16.6) | 0.058 |
| BCLC stage (B/C vs. 0) | 8.85 (1.97,39.68) | 0.004 | 4.29 (0.81,22.7) | 0.086 |
| CLIP stage (1 vs. 0) | 1.49 (0.86,2.56) | 0.153 | 1.21 (0.6,2.46) | 0.597 |
| CLIP stage (2-5 vs. 0) | 3.83 (2.28,6.45) | <0.001 | 1.38 (0.5,3.84) | 0.534 |
| *ALDOA* (high vs. low) | 1.39 (1.11,1.74) | 0.004 | 1.21 (0.93,1.56) | 0.154 |

**Notes:** AFP: alpha-fetoprotein, ALT: alanine aminotransferase, TNM: tumor-node-metastasis, BCLC: Barcelona Clinic Liver Cancer, CLIP: Cancer of the Liver Italian Program, CI: confidence interval.

**Table. S7. Univariate and multivariate Cox regression analysis of overall survival in the Cancer Genome Atlas Liver Hepatocellular Carcinoma (TCGA-LIHC) datasets**

| **Variable** | **Univariate analysis** | | **Multivariate analysis** | |
| --- | --- | --- | --- | --- |
|  | **Hazard ratio (95% CI)** | ***p*-value** | **Hazard ratio (95% CI)** | ***p*-value** |
| **Age (≥ 65 vs. < 65)** | 1.24 (0.88,1.75) | 0.225 | - | - |
| **Gender (Female vs. Male)** | 1.21 (0.85,1.73) | 0.292 | - | - |
| **Family history of cancer (Yes vs. No)** | 1.18 (0.82,1.71) | 0.374 | - | - |
| **TNM stage (II vs. I)** | 1.43 (0.88,2.33) | 0.153 | 0.99 (0.52,1.88) | 0.976 |
| **TNM stage (III vs. I)** | 2.76 (1.81,4.23) | <0.001 | 1.89 (1.1,3.23) | 0.020 |
| **TNM stage (IV vs. I)** | 5.73 (1.77,18.58) | 0.004 | 4.64 (1.39,15.43) | 0.012 |
| **Histologic grade (G3-4 vs. G1-2)** | 1.12 (0.78,1.62) | 0.529 | - | - |
| **Ishak score (5-6 vs. 0-4)** | 0.82 (0.48,1.4) | 0.465 | - | - |
| **Child-Pugh grade (B-C vs. A)** | 1.63 (0.81,3.3) | 0.172 | - | - |
| **Vascular invasion (Micro vs. none)** | 1.21 (0.77,1.9) | 0.404 | 0.96 (0.57,1.63) | 0.889 |
| **Vascular invasion (Macro vs. none)** | 2.49 (1.13,5.49) | 0.024 | 1.93 (0.84,4.4) | 0.119 |
| **AFP (positive vs. negative)** | 1.66 (1.08,2.57) | 0.022 | 1.46 (0.9,2.37) | 0.123 |
| **Residual tumor (R1-2 vs. R0)** | 1.68 (0.82,3.45) | 0.159 | - | - |
| ***ALDOA* (high vs. low)** | 1.28 (1.11,1.48) | 0.001 | 1.22 (1.03,1.45) | 0.025 |

**Notes:** TNM: tumor-node-metastasis, AFP: alpha-fetoprotein, CI: confidence interval.
